# Museomics for reconstructing historical floristic exchanges: Divergence of stone oaks across Wallacea

**DOI:** 10.1371/journal.pone.0232936

**Published:** 2020-05-22

**Authors:** Joeri S. Strijk, Hoàng Thi Binh, Nguyen Van Ngoc, Joan T. Pereira, J. W. Ferry Slik, Rahayu S. Sukri, Yoshihisa Suyama, Shuichiro Tagane, Jan J. Wieringa, Tetsukazu Yahara, Damien D. Hinsinger

**Affiliations:** 1 State Key Laboratory for Conservation and Utilization of Subtropical Agro-bioresources, Guangxi University, Nanning, Guangxi, China; 2 Biodiversity Genomics Team, Plant Ecophysiology & Evolution Group, Guangxi Key Laboratory of Forest Ecology and Conservation, College of Forestry, Guangxi University, Nanning, Guangxi, China; 3 Alliance for Conservation Tree Genomics, Alliance for Conservation Tree Genomics, Pha Tad Ke Botanical Garden, Luang Prabang, Laos; 4 Faculty of Biology, Dalat University, Dalat, Vietnam; 5 Sabah Forestry Department, Forest Research Centre, Sandakan, Sabah, Malaysia; 6 Environmental and Life Sciences Programme, Faculty of Science, Universiti Brunei Darussalam, Gadong, Brunei Darussalam; 7 Kawatabi Field Science Centre, Graduate School of Agricultural Science, Tohoku University, Miyagi, Japan; 8 The Kagoshima University Museum, Kagoshima University, Kagoshima, Japan; 9 Naturalis Biodiversity Center, Leiden, The Netherlands; 10 Center for Asian Conservation Ecology, Kyushu University, Fukuoka, Japan; 11 Génomique Métabolique, Genoscope, Institut de Biologie François Jacob, Commissariat à l′Énergie Atomique (CEA), CNRS, Université Évry, Université Paris-Saclay, Évry, France; Museum National d'Histoire Naturelle, FRANCE

## Abstract

Natural history collections and tropical tree diversity are both treasure troves of biological and evolutionary information, but their accessibility for scientific study is impeded by a number of properties. DNA in historical specimens is generally highly fragmented, complicating the recovery of high-grade genetic material. Furthermore, our understanding of hyperdiverse, wide-spread tree assemblages is obstructed by extensive species ranges, fragmented knowledge of tropical tree diversity and phenology, and a widespread lack of species-level diagnostic characters, prohibiting the collecting of readily identifiable specimens which can be used to build, revise or strengthen taxonomic frameworks. This, in turn, delays the application of downstream conservation action. A sizable component of botanical collections are sterile—thus eluding identification and are slowing down progress in systematic treatments of tropical biodiversity. With rapid advances in genomics and bioinformatic approaches to biodiversity research, museomics is emerging as a new field breathing life into natural collections that have been built up over centuries. Using *MIGseq* (multiplexed ISSR genotyping by sequencing), we generated 10,000s of short loci, for both freshly collected materials and museum specimens (aged >100 years) of *Lithocarpus*—a widespread tropical tree genus endemic to the Asian tropics. Loci recovery from historical and recently collected samples was not affected by sample age and preservation history of the study material, underscoring the reliability and flexibility of the *MIGseq* approach. Phylogenomic inference and biogeographic reconstruction across insular Asia, highlights repeated migration and diversification patterns between continental regions and islands. Results indicate that co-occurring insular species at the extremity of the distribution range are not monophyletic, raising the possibility of multiple independent dispersals along the outer edge of Wallacea. This suggests that dispersal of large seeded tree genera throughout Malesia and across Wallacea may have been less affected by large geographic distances and the presence of marine barriers than generally assumed. We demonstrate the utility of *MIGseq* in museomic studies using non-model taxa, presenting the first range-wide genomic assessment of *Lithocarpus* and tropical Fagaceae as a proof-of-concept. Our study shows the potential for developing innovative genomic approaches to improve the capture of novel evolutionary signals using valuable natural history collections of hyperdiverse taxa.

## Introduction

Natural history collections represent a spatio-temporal window on life on earth, allowing us to study the evolution of biological diversity [1–2]. They are therefore not merely a collection of static objects, but offer a vast source of information on the origin and functioning of biodiversity and the mechanisms underlying evolutionary diversification awaiting scientific inquiry [3–4]. Herbaria are especially useful in biogeography, as they provide spatio-temporal records for taxon occurrences at both global [5] and local scale (see for example [6]. In an era where biodiversity (and its continued persistence) is increasingly under threat, biological collections are also vital as a baseline for long-term studies [7]. Research involving permanent plot systems and active biomonitoring of sites, allows us to better understand and mitigate the effects of our actions on biological communities and the environment. None of these are possible without extensive and well-curated natural history collections which are allowed to grow and be actively worked upon by the scientific community [1,8–10]. Natural history collections also offer an extensive resource base for raising public awareness and promote opportunities to involve younger generations, non-specialists and private organizations in a community drive to categorize and describe all living organisms (e.g. citizen science, school programs, online digitization) [11–12]. Like natural history collections, tropical tree diversity (estimated to number over 60,000 spp.; [13–14]) is a treasure chest holding vast amounts of biological and evolutionary information. Unfortunately, straightforward access for scientific study to either, requires the consideration and resolution of several major theoretical and practical challenges [15–17]. In the case of museum collections, the main obstacles are related to the fragmented state of DNA material and the sterile state of many field collected specimens (e.g. [18], lists numbers in permanent plot studies). Extensive species ranges, fragmented knowledge of tropical tree diversity and phenology, as well as a widespread lack of species-level diagnostic characters prohibit collecting readily identifiable specimens which can be used to build, revise or strengthen taxonomic frameworks. Generating genome-level data from either source remains challenging for both practical and conceptual reasons.

### Study system

Among biologists, island systems have long been favoured as the optimal models to study evolution and evolutionary divergence. The Malesian biogeographical region, situated between the core Asian and Australian continental mainland, constitutes one of the world's geologically most complex regions making it of exceptional interest for biogeography [19]. The rich plant (and animal) communities of Malesia (now subdivided in Malesia and Papuasia *sensu* [20]) are a patchwork of lineages resulting from exchanges between Sunda and Sahul, beginning from the late Oligocene [21–22]. The intervening region between the Sunda and Sahul shelves (*Wallacea*) and Wallace’s Line (separating Wallaceae from Sunda), mark a major zoogeographic barrier and were first explored and documented by A.R.Wallace [23–24]. Their exact demarcation, the historical transition of the region, and the ability of lineages to traverse them, have been studied for a wide range taxa and periods, showing that major differences existed (and continue to exist) for each, in plants as well as animals [25–27]. Extensive geological diversity, coupled with historical discontinuous connectivity, have had lasting consequences on the ability of lineages to traverse, persist and evolve in the intermittent region over geological time. The transition of terranes (and subsequent orogeny) resulted in the presence of shifting barriers and pressures for biota to disperse, exchange and escape (e.g. PARLs in [28]; birds in [29].

The forest communities of Asia are unique and distinct from those in Africa and the Neotropics, by being particularly speciose in a number of tree families (e.g. Dipterocarpaceae, Myrtaceae, Lauraceae and others [30]). Not surprisingly perhaps, few families with large seeds [i.e. those with many species bearing seeds too large for birds and/or with traits connecting them to specific dispersing terrestrial elements like scatter hoarding rodent fauna] are present throughout the entire region and shared between climatic zones. One such family is Fagaceae (~700 Asian species in six genera), occurring from coastal and lowland conditions to over 4000m in the Himalayan region. The genus *Lithocarpus* Blume, with over 339 accepted species, is the second largest genus in the family and endemic to Malesia and the greater Southeast and East Asian region [31–35]. Previous studies on the genus using standard chloroplast markers and ITS for 48 species, identified two geographic hotspots of species diversity [Borneo and Indochina; [36–37]]. Other studies have focused on the influence of life history traits on genomic diversity [38] and the evolution of the ‘*enclosed receptacle*’ (ER) fruit type as a defense mechanism for seed predation [39–40].

These studies provide important insights on *Lithocarpus*, but the included molecular data and geographic coverage were limited. Because of DNA conservation issues they were also restricted to species for which fresh or recently collected materials were available and for which fruiting materials were available. The generic classification, by now outdated and incomplete, still relies on the original work done by Camus [41] and is entirely based on morphological characters of which the validity and utility have been hard to verify. Relatively little is known about genomic diversity (or its variability at various taxonomic and geographic scales) and evolutionary history throughout the larger region. Evolutionary links between continental species and those occurring in very distant and undersampled regions (e.g. parts of Indonesia, the Philippines, New Guinea) are particularly understudied. Specifically, the rate and mode of spread of this continental genus throughout the cline of archipelagoes that stretches between the Asian mainland and the Australian continent needs further clarification [30].

The geographic range of the genus is extensive and obtaining a representative sample set of material is both time consuming and costly. However, *Lithocarpus* specimens have been widely collected during the biological explorations of the 19th-20th century and large quantities are available in natural history collections, making the genus an ideal candidate for historical genomics or “*museomics*”.

Museomics has recently emerged as a promising approach to obtain a relatively high volume of informative genetic data from specimens in natural history collections [42–44] or from subfossils and archaeological remains [45], owing to the specific requirements of Next Generation Sequencing (NGS), in particular those of the Illumina platform. This platform is characterized by resulting in short reads (~150bps) from DNA fragments ~250-500bps long, rendering the output optimal for library construction and negating the previous requirement of high molecular weight DNA. This has been of particular interest for obtaining portions of the nuclear genome from herbarium specimens (e.g. by applying whole genome sequencing of model species, in-solution enrichment by hybridization, RADseq approaches or by resequencing degraded samples and mapping on a reference genome)(e.g. [46–48]). Also, low depth sequencing of the nuclear genome (ca. 10X) has been used to assemble coding regions, including the adjacent regions in species with low-complexity genomes. However, a Whole Genome Shotgun (WGS) approach still remains difficult and expensive for biodiversity and evolutionary studies, where commonly dozens to hundreds of samples are involved. Also, a RADseq approach, based on digestion of high-molecular weight DNA, is usually unsuitable for herbarium samples that commonly have short (< 500bp) or very short (<200bp) fragments. RADseq is also sensitive to DNA degradation, especially to deamination at fragment ends [49]. However, recent development of this approach, including the use of a second restriction enzyme (e.g. in the ddRADseq protocol) and/or the retrieval of RADseq-like capture-based enrichment of those loci in historial material (e.g. the HyRAD protocol) have proved useful in some evolutionary studies (i.e. younger specimens <25 y.o. [50]). Despite enrichment of targeted loci *prior* to sequencing by in-solution hybridization has been proven useful when using herbarium specimens [46,51–53], it remains expensive and relies on building an initial DNA library (usually built for the Illumina platform). Constructing such a library can be challenging in cases where DNA degradation is severe or only small amounts can be obtained (e.g. as is the case when using herbarium collections).

In 2015, a novel solution was proposed using “multiplexed ISSR genotyping by sequencing” (*MIGseq* [54]) to generate hundreds of genome-wide loci from suboptimal sources of DNA (e.g. silica gel dried leaves samples or herbarium specimens, but more recently also applied to dried insects [55]). The method relies on PCR amplifications that generate both the loci and sequencing libraries in two steps, remaining relatively inexpensive and easily mastered by any individual with basic molecular laboratory training. Loci are generated by multiplexed amplification of ISSR regions (Inter Simple Sequence Repeats) using a set of SSR-specific primers, followed by ligation of Illumina sequencing adapters in a second amplification step. The libraries are then ready to sequence on an Illumina platform. Because SSRs are widespread in each genome and their location is relatively conserved at generic levels, short orthologous regions (~300–800 bps) can be generated for any set of taxa [54].

In addition, the *MIGseq* protocol, based on PCR amplification steps, allows for low DNA quantity starting material, as well as degraded DNA. Indeed, despite deamination being a commonly observed feature in historical material, PCR steps amplify internal sequences of the DNA fragments, leaving their extremities not sequenced. Because deamination occurs mostly at fragment extremities, the *MIGSeq* approach removes this issue.

With *Lithocarpus* selected as our study group, all the before-mentioned conditions (i.e. widespread in a biogeographically complex region; large numbers of collections; complex morpho-character system; incomplete evolutionary and taxonomic framework) were met. Combining museomics and bioinformatics our aim was two-fold: 1) to assess the potential of *MIGseq* sequencing for museomics using a widespread and heavily collected genus; 2) to generate novel genomic insights in species relations, genomic diversity and divergence for *Lithocarpus* spanning the full distribution range by including a representative set of taxa.

## Results and discussion

### *MIGseq* and bioinformatic approach

A total of 94,944 loci were generated in this study, of which 60,997 were filtered out due to low taxa sampling (i.e. they were found in less than 4 species). 42,060 loci were filtered out due to a number of alleles >2. Following the filtering process using *ipyrad*, 7,371 loci were retained. The highest and lowest number of reconstructed loci were found in *L*. *sootepensis* (BGT1034–1635 loci) and *L*. *aspericupulus* (BGT3281, 66 loci). The distribution of the length of the loci against the number of species sampled showed no clear correlation (S5 Fig). Comparing with case studies reported in the original *MIGseq* publication, our sampling included a mix of herbarium and silica-dried samples and resulted in a roughly similar number of loci [54]. Using STACKS, an approach more adapted to population-level inquiries [56], Fagaceae-focused studies retrieved many more loci [57–58]. However, here we opted for a conservative approach in this complex group, to avoid inclusion of paralogs in our dataset.

Analyses of the DNA degradation of the assembled *MIGseq* loci with mapDamage did not show the usual pattern of deamination at the 5’ and 3’ ends of fragments (e.g. [49]), neither for each sample (S7 Fig) nor when considering herbarium and silica samples separately (S8 Fig). Indeed, *MIGseq* does not use the actual DNA fragments, but amplified ones through the two PCR steps, resulting in consensus sequences that are damage free [49]. Although this could in theory lead to a bias in fragment representation, it is very unlikely for the SSR sequence (where the primers bind during amplification) is damaged in all DNA fragments in the DNA samples. Therefore, we do not expect a qualitative change in the loci pattern (i.e. no change in the presence-absence of fragments), but cannot exclude the option that a few fragments were lost due to a lower level of amplification (and were thus removed from the results during the filtering steps). However, the overall absence of damage patterns in our results suggest that *MIGseq* could be a useful and elegant solution to avoid inclusion of DNA damage in phylogenetic datasets, although further investigation remains necessary to confirm this.

The location of *MIGseq* loci. assessed by mapping them on the genome of the common oak, showed no specific pattern and a distribution of distances showed a global Poisson distribution corresponding to a random distribution (S9A Fig). Of the total 7,371 loci, only 19 could not be mapped on the common oak genome, reflecting the high level of conservation of the genomes in Fagaceae. An average of 9.31 loci per Mb (min-max: 7.80–10.73; st.dev: 0.88) were found in the common oak genome, showing an expected strong linear correlation between the chromosome length and the number of *MIGseq* loci (r^2^ = 0.995). 464 of the remaining loci were mapped near CDS (i.e. coding regions), while 2,258 loci mapped near Transposable Elements (TE), 61 and 25 near the 5’ UTR and 3’ UTR regions, respectively (S10 Fig). Considering the usual observed linkage disequilibrium in oaks (i.e. <100-400bp—see [59–60], the majority of loci should not be influenced by selection (S9B and S9C Fig). Reflecting the high proportion of TE-elements in the common oak genome (52%—[61], a large part (31%) of the loci were found close from TEs, despite only ~100 being directly located within TE.

### Phylogenomics and molecular dating

**Fig 1:**
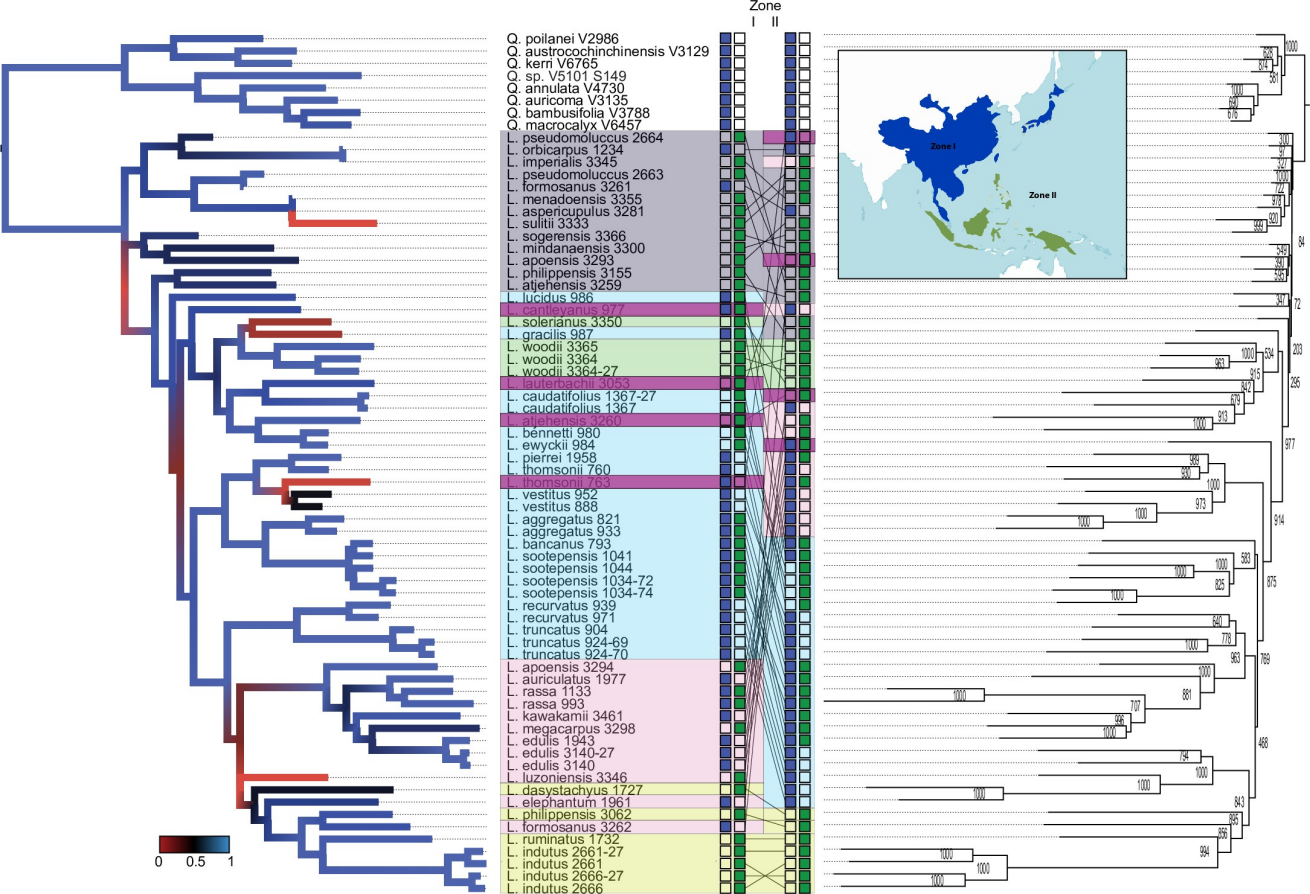
**Comparison of PhyML results obtained from SNPs dataset (left) with the NJ tree (right) obtained using loci presence-absence data.** Inset map shows the global distribution of *Lithocarpus* (blue+green) in reference to continental (blue) and insular (green) datasets. Clade colors and connecting lines indicate alternate placements of species in opposing trees. Numbers (right) and colors (left) on branches indicate nodal support values.

Results of our phylogenetic analyses using PhyML and presence-absence (using a NJ approach) resulted in slightly incongruent trees with good support for most major clades (Fig 1). Both *Quercus* and *Lithocarpus* are recovered as monophyletic groups. Overall, phylogenomic analyses of *MIGseq* data result in a well supported reconstruction of evolutionary relationships.

Patterns of incongruence are restricted to the placement of major clades and do not affect the inferred relationships of individual species. Two polytomies at higher levels raise additional questions on the identification of some specimens, and the broader relationships within the genus.

Technical testing confirmed the robustness of the *MIGseq* protocol, with duplicated conspecific individuals grouping together in both the PhyML and loci tree, including those species represented by both herbarium- and silica-based collections (e.g. *edulis*, *indutus*). This underscores the resolving power and the applicability of the *MIGseq* approach to a wide range and age of template materials, and material subjected to a range of collecting, preservation and storage treatments.

The NJ-tree reconstructed using presence-absence of retrieved loci differs in overall outline, but is consistent in the reconstruction of major lower clades. Support for most branches is high, but here, branches lower in the phylogeny receive consistently lower support values. This could be due to artifacts resulting from DNA degradation or the limited sample size of our study—not all retrieved loci had matches in other or conspecific taxa. The ability to increase sample and species coverage by adding from existing natural history collections can resolve this issue quickly, contrary to traditional studies which are field collection based and generally limited in available material. The inclusion of freshly sampled field material for a number of species spread throughout the tree could result in a strengthening of the backbone of the phylogeny due to the higher numbers of loci that are retrieved, and a higher number of loci from ‘*museum*-species’ that can be matched as a result.

We selected species from across the distribution range of the genus, sampling subsets restricted to islands, the continental mainland and those species occurring on both. Analyses recovered clades reflecting this geographic background, with some only displaying island taxa (*atjehensis*-*bennettii*-*ewyckii*), while others only contain species from mainland Asia (*pierrei*, *thomsonii*-*vestitus*) (Fig 1, S1 and S2 Figs). The joint clustering of continental and island species seems more apparent though, with at least two clusters resulting in sister clades that are completely continental and mixed. The majority of clades show species with a mix of distribution ranges, suggesting that historical exchange and dispersal between zones III-VI (and between zones III and IV, see S1 and S2 Figs) have been more common than previously assumed [30].

**Fig 2:**
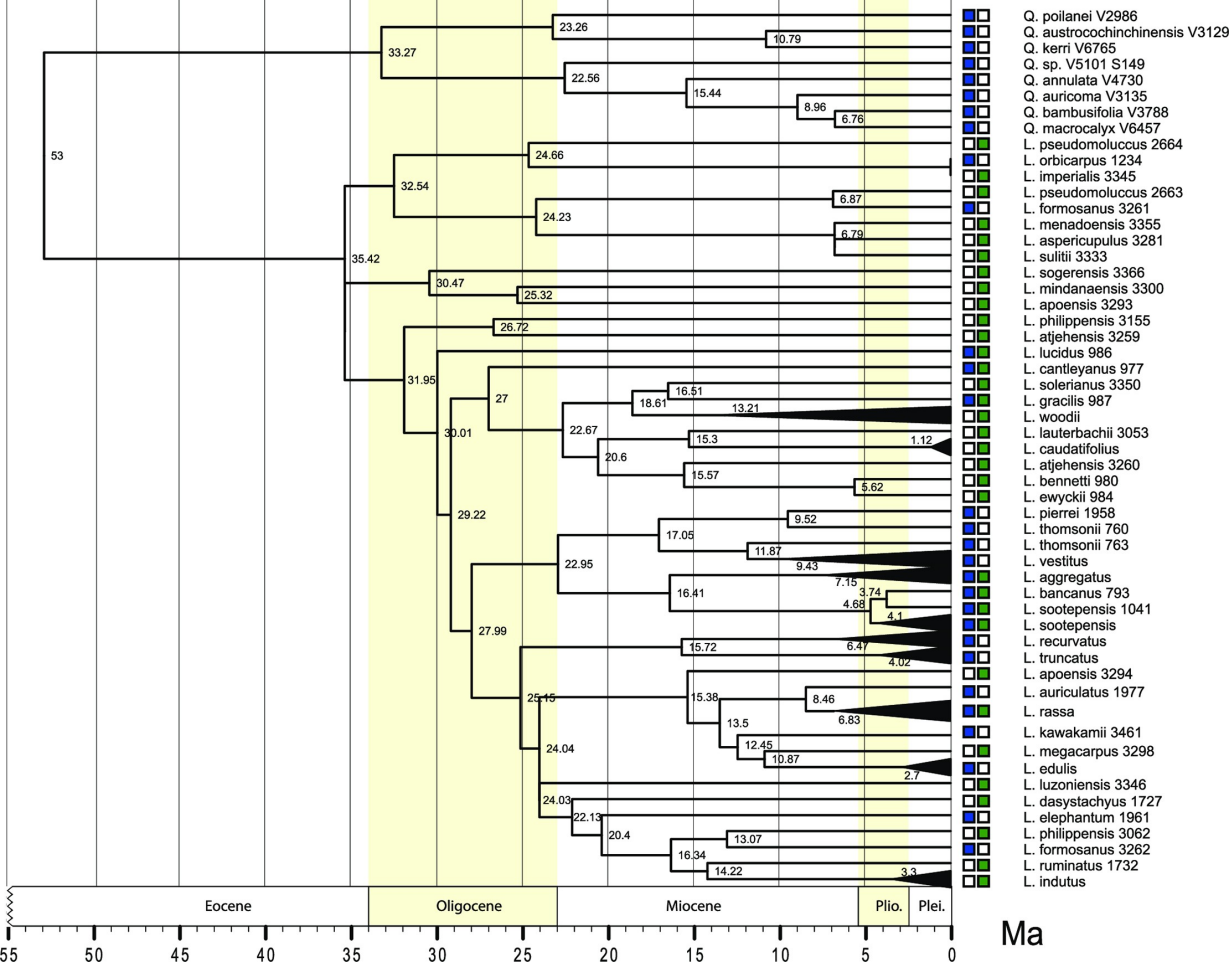
Dated tree based on the reconstructed *MIGseq* loci (Penalized likelihood). Nodes ages (in Ma) are indicated as well as the geographic range in the 2-zones configuration (tips of the branches, colors according to those in Fig 1).

The results of the molecular dating analyses show the first age estimates for Wallacean and Sahul *Lithocarpus* (Fig 2). Bearing in mind that performing molecular dating analyses on undersampled taxon sets can adversely affect node age estimates, a conservative approach to interpreting these initial results is essential here. However, the crown age estimate for *Lithocarpus* is roughly in line with node age estimates and attributed fossil ages for the genus reported elsewhere [62]. Age estimates for underlying clades can be expected to change with the inclusion of additional species. Patterns of higher clade divergence hint that there may have been successive waves of cross-regional diversification (Early Oligocene; Early-Mid Miocene; Late Miocene-Pliocene (Fig 2), which would also be in line with expected patterns resulting from repeated expansion and contraction of forests in the region in response to glacial oscillations (but see ([63–65]). Additional species and molecular data will be needed to enable more comprehensive dating analyses and ancestral range reconstructions at finer geographic scales.

Of interest to note is the position of the five species endemic to New Guinea. These species appear widely spaced and never cluster together in our analyses, suggesting multiple independent arrivals to the most eastern part of the distribution range of the genus. Furthermore, it appears that *Lithocarpus* on New Guinea have different source origins, with some having close affinities with Wallacea-Malesia (*lauterbachii*, *aspericupulus*), while others suggest close relationships with Philippine island species (*sogerensis*) and Japan (*megacarpus*). If corroborated with more extensive data, these patterns would confirm the ability of large seeded trees to migrate along the eastern frontier of Wallacea and Malesia. Such patterns have been previously shown to exist in freshwater invertebrates and birds dispersing across the Philippine Islands to as far south as Fiji, in relatively recent times (25–12 Ma; [66–69]).

Across its range, *Lithocarpus* occurs from lowland to mid-elevation, reaching their maximum levels of species diversity in the latter. Although a range of source origins for *Lithocarpus* is proposed here, this pattern appears to be repeated in New Guinea, where most species ranges are restricted between 900–1800 (except for species like *L*. *vinkii*, that appear to be wholly confined to <500 m elevation). This would be in line with regional patterns of lineage exchange across Wallace’s Line, where most of the lowland New Guinea flora appears to be of Sunda origin and the highlands are predominantly populated with austral-Gondwanan lineages [28]. The proto-island scenario of New Guinea described in [29], where a multitude of islands of varying geological origin and age existed from the Cenozoic until about 5 Ma, allows for the range of nodal age-estimates obtained here for the arrival of various *Lithocarpus* lineages on New Guinea. The significance of this configuration for the divergence (both locally and globally) of a host of other taxa has previously been documented [70–71], and indeed, the consensus now is that species-level divergence in major groups in New Guinea is recent (<5 Ma). This is supported by increasingly detailed paleotectonic data and geological evidence that dates substantial landmass formation to <10 Ma [72–73]. Moreover, timing and sequence of (specific) collision events and their impact on the evolution of a host of other faunal which dispersed across the region is increasingly well documented [29,74–75]. Following formative studies by Hall [72–73,76] on the geological evolution of the region, [30] recently published a synthesis on the historical assembly of the flora of the region, highlighting enduring gaps in our understanding. With these challenges now defined in a clear spatio-temporal framework and their resolution being well within reach of genomic applications, it is only a matter of time before major plant taxa will be treated in a similarly detailed approach as is rapidly becoming the new standard for major faunal groups.

Our observations suggest that dispersal of large seeded trees like *Lithocarpus* (and quite possibly other Fagaceae like *Castanopsis*) throughout Malesia and across Wallacea has been a commonly recurring event, despite large geographic distances and the presence of water barriers. Irrespective of the age of our sample material, interesting biogeographic signals are present in the retrieved data. This confirms the power of the *MIGseq* approach for museomic applications and suggests that innovative analytical methods could be developed further to improve the capture of evolutionary signals from valuable herbarium specimens.

### Museomics and regional genomic diversity

**Fig 3:**
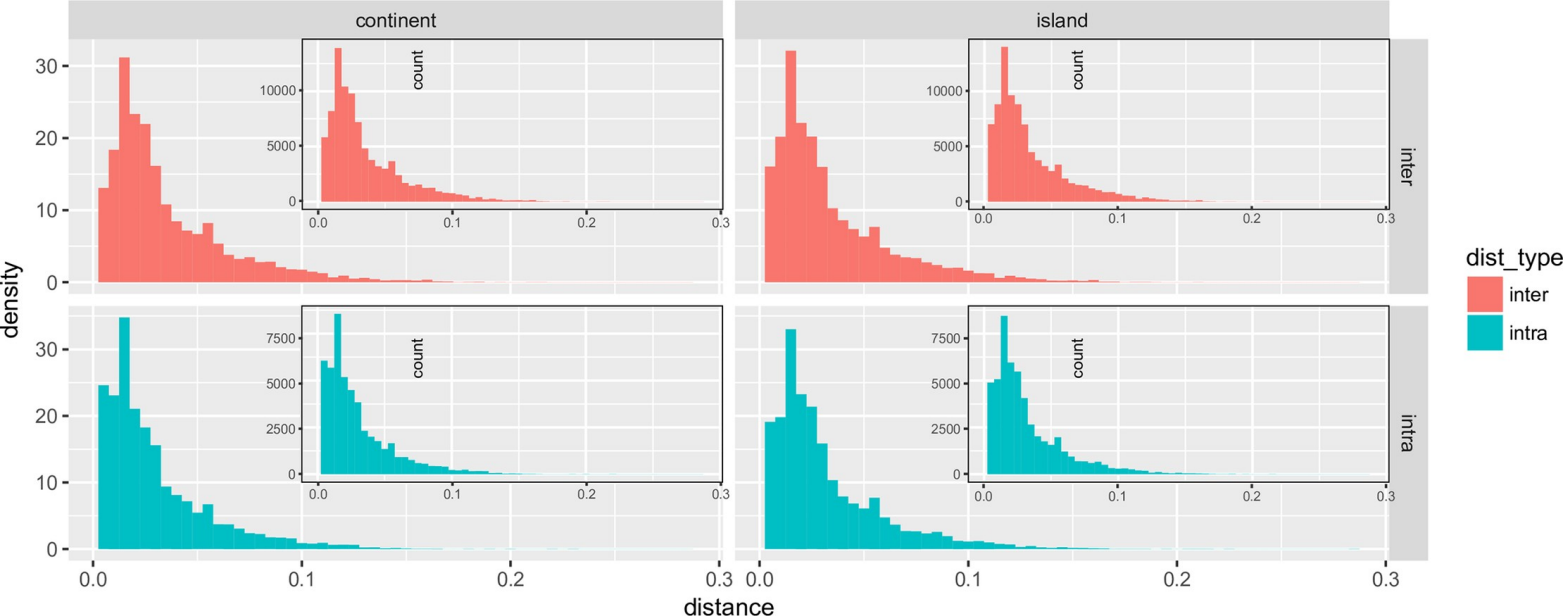
Density histogram of inter- and intra-zone distances according to study region (“continent” or “island”). Inter-zone distance: red; intra-zone distance: blue. Species found in both continental and island areas were excluded. For each plot the corresponding count histogram is plotted as inset.

The comparison of intra- and inter-zone genomic distances for the continent *vs* island configuration showed a higher proportion of closely related species (i.e. small genomic distances) in the continental species, with smaller median values than in islands (0.025 vs 0.026 and 0.023 vs 0.025 for the inter- and intra-zone continental vs island comparison, respectively). This is especially apparent when comparing intra-zone genomic distances (Fig 3). The two-factor Anova analyses using *genomic distance types* (intra- *vs* inter-zone configuration) and *geographic zones* as explicative variables of genomic distance were highly significant (p < 2.2 x 10^−16^) for all comparisons, except for the general distances distribution between continental and islands species (2 Zones–Fig 3; 4 Zones–S3 Fig; 6 Zones–S4 Fig). This suggests that different evolutionary processes and events are underlying continental species and island species diversities. One of these factors could be the patchy structure of the island environment contrary to that of a continuous landmass the continental species experienced during their diversification. Another factor could be the effect of population size fluctuations and population connectivity over time. To test this further, population level sampling across the range would be needed for a large number of species—something that is possible using museomics and the *MIGseq* approach, but beyond the scope of this study.

Nearly one third of the samples included in this study originated from *museum* collections (ranging from 17–109 years old) and all were collected on non-continental sources. Silica samples ranged in age from 2–7 years old and varied in type of collection locality across Asia (islands and continental sites). According to our expectations, the average number of loci retrieved from silica collections (mean = 695.71) was slightly higher than those retrieved from herbarium collections (mean = 528.48), but not significantly different.

**Fig 4:**
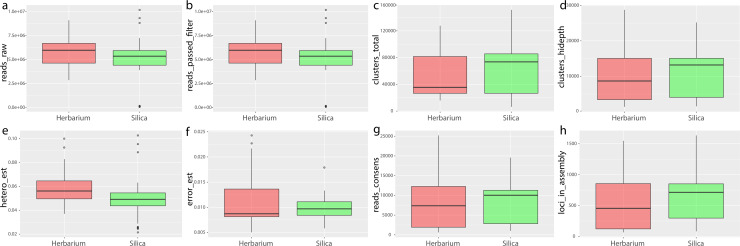
Box plot comparisons of herbarium and silica sample characteristics for statistics derived from ipyrad analyses. Herbarium material: left, red; silica materials: right, green (see also S6 Fig).

Difference detected in dataset characteristics (e.g. the number of both clusters and clusters retrieved with high depth, error level, the number of reads assembled in the consensus sequences, and the final number of loci assembled) between herbarium samples and silica-dried samples were negligible. However, we retrieved a slightly higher number of raw reads, filtered reads (p = 0.0169, Fig 4A and 4B) and heterozygosity level (p = 0.0169, Fig 4E) from herbarium material than from silica-dried samples.

The ages of sample material did not strongly correlate with any of the properties of the assembled dataset (maximum r^2^ = 0.13, negative correlation with the heterozygosity estimates) (S6 Fig). Correlation between the number of clusters reconstructed with high depth and both heterozygosity and error rates were low (r^2^ = -0.44 in both cases), as well as the correlation between error and heterozygosity estimates (r^2^ = 0.46), showing evidence that these values were not biased (S6 Fig). Increased heterozygosity is a common feature in studies based on degraded DNA, due to deamination at the terminus of degraded DNA fragments. However, no pattern of deamination was found in our data, neither in herbarium nor in silica samples (S7 and S8 Figs).

In line with our expectations, the final number of loci in the assembly was correlated with the number of reads mapped to the consensus sequence (r^2^ = 0.81), to the number of high depth clusters (r^2^ = 0.85) and to the total number of clusters (r^2^ = 0.86), but not to the numbers of both raw (r^2^ = 0.25) or filtered (r^2^ = 0.25) reads per sample (S6 Fig).

Previous studies demonstrated that the rate of DNA fragmentation in preserved silica-dried leaf material remains high for a short duration after desiccation, but decreases rapidly thereafter [42,77]. At present, such material is not suitable for complete genome-targeted studies, although they can be very useful for organellar-based research [44,78]. In studies applying methods such as *MIGseq*, which are aimed at amplifying and sequencing small DNA fragments from any genome in the cell (organellar or nuclear), the age and extent of fragmentation of genetic material in the tissue cells does not appear to have much effect on the number of loci obtained. Our oldest sample (collected on Mindanao, Southern Philippines in 1909) still resulted in 506 loci (S1 Table, S5 Fig). Thus, museum-oriented studies, which by default deal with large numbers of specimens subjected to variable collection methods, historical preservation treatments and of widely ranging ages can benefit from flexible NGS approaches like *MIGseq*. This is particularly poignant, as these methods and treatments (e.g. chemical/physical desiccation, storage in fluids, freezing) are generally not documented on specimens or labels and are known to adversely affect traditional approaches in DNA sequencing [79–80].

Although this is a preliminary study, these are exciting first insights into the evolution and biogeographic divergence across the range of *Lithocarpus*. Scaling up the sampling will be a logical next step to boost clade support, clarify the position of smaller basal clades and to clear up the relationships between island assemblages and their ancestral ranges.

## Conclusions

Natural history collections are storehouses of evolutionary and biological information, constituting exceptional historical investments of time, money and human endeavor spanning centuries [81]. The world’s 3400 herbaria hold an estimated 350 million samples, collected over the last 400 years [82]. With the rapid development of museomic and novel bioinformatic approaches to process large genomic datasets from a wide range of sources [83], the use of specimens that could not be collected nowadays for ‘spatial’ reasons [84] can address evolutionary questions (e.g. this study), resolve taxonomic placements of extinct taxa [78], or construct a biodiversity catalogue of an entire flora (e.g. the PhyloNorway project, which aims to build a reference database for all Norwegian vascular plants). In addition to the use of herbarium specimens as representatives of their inaccessible ‘present day’ counterparts, museomics also allows to directly address the evolution of species through dated collections. For example, historical herbarium specimens of sweet potato (*Ipomoea batatas*) have been used to highlight the replacement of native varieties by introduced types, demonstrating directly (i.e. not inferred from modern samples) the variation of genotypes through time [85]. Museomics is still an emerging field with many challenges to overcome, especially in botany where herbarium DNA decays up to six times faster than in bones [77].

## Materials and methods

### Taxon sampling

We designed our sampling to be geographically balanced, covering the entire distribution range of *Lithocarpus*, selecting species restricted to insular Asia (22 spp.), exclusive to the Asian continental mainland (10 spp.), and occurring on both islands and mainland (7 spp). Leaf material was then collected from herbarium collections for 17 species of *Lithocarpus* representing most corners of the insular distribution range of the genus (S1 Table and map inset in Fig 1). As these materials were collected over the past century (up to ~100 years ago), most of these contain only rough locality data and no exact geographical coordinates (See S1 Table).

We selected leaf material for an additional 22 species acquired through fieldwork/sampling of living collections in botanical gardens and forest areas: 1) Thailand—Department of National Parks, Wildlife and Plant Conservation (vouchers lodged at BKF); 2) Laos—National University of Laos (vouchers lodged at KYO); (MOU between Kyushu University and Faculty of Forestry, National University of Laos (FOF); 3) Indonesia—Herbarium Bogoriense, Lembaga Ilmu Pengetahuan Indonesia (LIPI) (vouchers lodged at BO); 4) Singapore–NParks, Singapore Botanical Garden (vouchers lodged at SING and BGT), research and collecting permits issued; 5) Sabah, Malaysia–Forest Research Center (vouchers lodged at FRC and BGT); 6) Cambodia—Forest Administration of Cambodia (vouchers lodged at National Herbarium); Japan—Kagoshima University (vouchers lodged at KAG); 7) France–Arboretum de Passadou (with kind permission of the owner J.L.Hélardot; vouchers lodged at BGT); 8) Vietnam—Dalat University (vouchers lodged at DLU), research and collecting permits issued).

On a global scale, our sampling covers 11% of all species described. All vouchers of fresh materials were deposited at the Biodiversity Genomics Team (BGT) herbarium of Guangxi University. We used selected data from a previous study on *Quercus* to root the *Lithocarpus* ingroup [43]. All voucher and collection data are available in S1 Table.

#### DNA extraction and sequencing

Genomic DNA was purified from approximately 10 mg of leaf material using the Plant Genomic DNA kit (TIANGEN Biotech, Beijing, China), following the GP1 protocol. Following the *MIGseq* methodology and protocol [54], we amplified loci in a two-PCR step process. Our protocol followed standard conditions and described primer sequences, with the exception of a DNA starting amount of 50ng, and an increase in cycle number (increased to 27–30 cycles in the first PCR step, see below). Sequencing was performed by Novogene (Beijing, PR China) on an Illumina HiSeq-Ten-X, according to the manufacturer instructions. Although *MIGseq* was designed to be highly multiplexed in order to reduce costs, we chose to generate approximately 1Gb of sequences per sample, representing a multiplexing of approximately 96–100 individuals per lane. Indeed, we expected herbarium samples to generate suboptimal DNA quality, thus decreasing the yield of loci at sufficient sequencing depths to be assembled. By generating more data than originally required by the *MIGseq* protocol, we maximized the potential retrieval of loci in these degraded samples.

We performed two technical tests to assess the robustness of the *MIGseq* protocol, and applied our findings to adapt the protocol to suboptimal samples. The first step tested the robustness of the method to different amounts of starting DNA material (30ng and 50ng), using *L*. *sootepensis* (BGT 1034) and *L*. *truncatus* (BGT 924); the second step assessed the effect of increasing the number of cycles during the first PCR step to 27 and 30 cycles, using 5 individuals (*L*. *caudatifolius* BGT 1367, *L*. *edulis* BGT 3140, *L*. *indutus* BGT 2661, *L*. *indutus* BGT 2666, *L*. *woodii* BGT 3364).

Orthologous loci among species were reconstructed using ipyrad v3.1.2 [86], with parameters as follows: no restriction overhang, 50bps as max low quality base calls, a minimum assembly depth of 5 for both statistical and majority rule base calling, a maximum of 2 (*Lithocarpus* ssp. are diploids, thus a number of alleles >2 would indicate a reconstructed paralog), 5, 8, 40 for the maximum number of alleles per site, N and heterozygous positions in the consensus and indels per locus, respectively. Loci were called only if found in more than 4 species. In the original *MIGseq* protocol, the authors assembled the loci dataset with STACKS [56]. However, despite that ipyrad was designed for restriction-based methods, it is in fact more efficient for studies at larger taxonomic scales [86]. All loci were used for divergence analyses, biogeographic and phylogenomic reconstruction.

Because *MIGseq* loci are not based on direct sequencing of degraded DNA, but on the sequencing of PCR-enriched loci, we did not expect to observe degradation patterns often seen in ancient DNA studies. Indeed, the PCR steps imply that the primer regions are complete and the resulting amplicons have blunt-ends, making them less sensitive to deamination that would occur at both ends of the genomic DNA fragment. To verify our assumption, we used the MapDamage 2.0 pipeline [87] to map the Illumina reads against our assembled loci and score the DNA damage in the reads, with default parameters.

Linkage disequilibrium can distort phylogenetic signals if loci used for the reconstruction are located near coding regions experiencing a selective pressure which varies from other parts of the genome [88]. Therefore, we assessed the location of our loci in the genome. However, no *Lithocarpus* assembled genome is currently available to map our *MIGseq* loci. As the synteny among genomes has been shown to be relatively high between common oak and chinese chestnut genomes [89]—that are less related to each other than *Lithocarpus* with *Quercus*—we used the genome of the common oak (*Quercus robur*), that was recently fully assembled to chromosome level [61], as a rough substitute for mapping. Assembled *MIGseq* loci sequences (one sequence per locus) were mapped against the genome of *Quercus robur* (version PM1N - 12 pseudomolecules) using Bowtie 2 version 2.2.0 and default parameters. Annotations for coding regions and Transposable Elements (TE) were downloaded for the PM1N genome and used to calculate the distance between each mapped *MIGseq* locus and the closest feature, using the “*closest*” function of the bedtools v2.28.0. with both the coding regions and TE annotations, and then plotted in R v3.5.3. According to the available annotations of the common oak genome, we considered the following features: the coding regions (CDS), the 5’UTR, the 3’UTR, and the transposable elements (TE).

### Phylogenomic analyses and molecular dating

We reconstructed a Maximum Likelihood (ML) tree using PhyML 3.1 [90–91] based on the concatenated SNPs found in the assembled loci from ipyrad. The GTR+I+G model was selected, with 4 gamma rate categories. Nodal support was estimated using the SH-like approach [92], for which values have been shown to be as conservative as the commonly used non-parametric bootstrap values [93], but much faster to compute in larger datasets (>7,000 loci, >73,000 SNPs herein) [94]. We also reconstructed a NJ tree based on the presence-absence of loci matrix using the package *ape* in R 3.4 (R Core Team 2014) and estimated nodal support by generating 1000 bootstraps.

Divergence of each locus was assessed by calculating the pairwise *p*-distance, then combining all comparisons and scoring them as “*intra*-zone” or “*inter*-zone” (see below). We performed a two-way ANOVA, followed by a Tukey Honest Significant Difference test (Tukey HSD) to identify significantly different pairwise differences among 2 (I-II), 4 (I-IV) and 6 (I-VI) biogeographical zones. All statistical analyses were performed using R v3.4 [95].

Divergence times were estimated using the Penalized-Likelihood method (PL) implemented in treePL v.1.0 [96]. To calibrate the divergence of *Lithocarpus* from *Quercus*, we used two Eocene macrofossils attributed to *Lithocarpus*, namely *L*. *karasorianus* and *L*. *timensis* from the Fossil Plants database (http://fossilplants.info/index.htm). These two fossils represent well conserved leaves, allowing us to determine they likely represent already derived morphs. In addition to this direct evidence, an Eocene divergence is coherent with previous date estimates in the family (e.g. ([62]). Therefore, we set the minimum age of the divergence of *Lithocarpus* and *Quercus* to 33.9 Ma, with an additional maximum age of 53 Ma, corresponding to the maximum age of the fossil calibration used elsewhere [97] for the entire Fagaceae family. To estimate the smoothing parameter, we used a cross-validation, ranging from 100,000 to 0.001, and then set the value to 0.01, according to the lowest Chi-square value (11562.7). Raw reads were deposited to the ENA, under project number PRJEB34850. Alignments and trees were deposited in the Dryad repository (https://doi.org/10.5061/dryad.xd2547dc8).

### Biogeographical analyses

For this study, we postulate that *Lithocarpus* as we know it today, is of continental Asian origin and dispersed southward into Malesia and Papuasia with local radiations occurring repeatedly over time. The current centers of species diversity are in IndoChina and on Borneo. The fossil record for *Lithocarpus* is poor, with most reliable finds in Russia (Late Eocene, *L*. *timensis* [98], Germany (Oligocene, *L*. *saxonicus* [99]) and Abkhazia (Pliocene, *L*. *longifolia* and *L*. *palaeouncinata* [100]). In China, fossil remains of fruits and leaves have been found in deposits dated to the Eocene, Oligocene and Pleistocene [101–102]. A recent fossil discovery confirms the presence of *Lithocarpus* precursors in the Nanning basin (Guangxi province, southern China) by the Upper Oligocene (33.9–27.82 Ma) [103] at which time the local conditions resembled a modern day warm monsoonal climate.

To explore biogeographic patterns in *Lithocarpus* spp., we divided our geographical and species datasets into “*continent*” and “*island*” groups and compared whether significant differences in genomic divergence exist between continental and island taxa.

Secondly, using existing biogeographical units in the distribution range of *Lithocarpus*, we divided the area into a) a four zone configuration (Zone I (China, Japan), Zone II (Southeast Asia, India), Zone III (Malesia) and Zone IV (New Guinea)), and b) a 6 zone configuration (as in a) but with Zone III subdivided further according to the definition of the Malesian Floristic Subkingdoms [the Western, Southern and Eastern Divisions respectively, as identified in [104] (S3 and S4 Figs). New Guinea (and associated island chains) are considered separately here, following the latest classification [20]. Using these two classifications (4 vs 6 zones) we tested for differences in data clustering, geography, diversity and phylogenetic signal, in an attempt to better understand how marine barriers and terrestrial corridors have affected lineage dispersal and genomic divergence of *Lithocarpus* in insular Asia.

## Supporting information

S1 Table Accession and voucher information. (PDF)

S2 Table Results of the Tukey HSD comparisons tests for the 4 zones configuration (a) or 6 zones configuration (b); **: p<0.01 significance threshold.
(PDF)

S1 Fig Maximum likelihood (ML) tree for the 4 zones configuration, based on SNPs identified in the assembled *MIGseq* loci. SH-like nodes support values indicated on nodes. Color of the branches indicate support values. Geographic range indicated at the tips corresponding to the inset map (see text for details).
(PDF)

S2 Fig Maximum likelihood (ML) tree for the 6 zones configuration, based on SNPs identified in the assembled *MIGseq* loci. SH-like nodes support values indicated on nodes. Color of the branches indicate support values. Geographic range indicated at the tips corresponding to the inset map (see text for details).
(PDF)

S3 Fig Density histogram of inter- (red) and intra- (blue) zone distances according to the 4 zones configuration. For each plot the corresponding count histogram is plotted as inset.
(PDF)

S4 Fig Density histogram of inter- (red) and intra- (blue) zone distances according to the 6 zones configuration. For each plot the corresponding count histogram is plotted as inset.
(PDF)

S5 Fig Descriptive scatter-plot of the loci used in this study. Each point represents one reconstructed locus. Horizontal axis: number of species assembled for a given locus; vertical axis: locus lengths.
(PDF)

S6 Fig Correlogram of the dataset used in this study. Statistics are derived from ipyrad outputs. *Age*: calendar year of collection of the samples; *reads raw*: number of generated Illumina reads used as input for the loci reconstruction for a given species; *reads passed filter*: number of reads after ipyrad filtering steps; *clusters total*: number of clusters assembled for a given species; *clusters hidepth*: number of clusters with a assembly depth > 5; *hetero est*: heterozygosity estimate for each sample; *error est*: error rate estimate for each sample; *reads consens*: number of reads from a given species used to generate the consensus sequence of a loci; *loci in assembly*: final number of loci reconstructed for a given species.
(PDF)

S7 Fig DNA misincorporation (C>T and G>A) patterns of sequencing read data from 62 *Lithocarpus* samples. Patterns were obtained by using MapDamage v. 2.0.6. Y-axis denotes the number of reads containing a nucleotide change from the reference sequence, and x -axis denotes position along the DNA fragment. A) misincorporation patterns at 5’ ends for each sample; B) misincorporation patterns at 3’ ends for each sample.
(PDF)

S8 Fig DNA misincorporation (C>T and G>A) patterns of sequencing read data for herbarium (blue) and silica (red) samples. Patterns were obtained by using MapDamage v. 2.0.6. Y-axis denotes the number of reads containing a nucleotide change from the reference sequence, and x -axis denotes position along the DNA fragment. A) misincorporation patterns at 5’ ends for each sample; B) misincorporation patterns at 3’ ends for each sample.
(PDF)

S9 Fig Distances between the *MIGseq* loci and genomic features from the PM1N oak genome. TE: Transposable Elements; 5prime: five prime Untranslated Transcribed Region; 3prime: three prime Untranslated Transcribed Region; CDS: Coding Regions. A) Distribution for all *MIGseq* loci; B) Distribution for *MIGseq* loci located closer than 5kb from a genomic feature; C) Distribution for *MIGseq* loci located closer than 1,000bp from a genomic feature.
(PDF)

S10 Fig Genomic location of the 7371 *MIGseq* loci. TE: Transposable Elements; 5’ UTR: five prime Untranslated Transcribed Region; 3’ UTR: three prime Untranslated Transcribed Region; CDS: Coding Regions; Others: not found in annotations from the PM1N oak genome.
(PDF)
