# Supplementary material for: Museomics for reconstructing historical floristic exchanges: Divergence of stone oaks across Wallacea
Source: PLoS One. 2020 May 22;15(5):e0232936. doi: 10.1371/journal.pone.0232936 (PMC7244142; doi:10.1371/journal.pone.0232936)
Supplement: S1 Table — (PDF) [file pone.0232936.s001.pdf]

Supplementary Table S1. Accession list and voucher information

1 All geographic information obtained from (Govaerts *et al.* 2008)

2 Data submitted to DRYAD, available at ######

Species	BGT database # and source material	Voucher information	Geographic distribution[1]	Dryad reference number[2]
Lithocarpus aggregatus Barnett	821 Silica	Chamchunroon 5181 (BKF), Chiang Mai, Thailand (2011)	Indo-China to Pen. Malaysia 41 THA VIE 42 MLY	######
Lithocarpus aggregatus Barnett	933 Silica	Sirimongkol 424 (BKF), Khamphaengphet, Thailand (2012)	Indo-China to Pen. Malaysia 41 THA VIE 42 MLY	######
Lithocarpus apoensis (Elmer) Rehder	3293 Herbarium	Price & Hernaez 745 (L), Samar, Philippines (1975)	Philippines 42 PHI	######
Lithocarpus apoensis (Elmer) Rehder	3294 Herbarium	Jacobs 8000 (L), Luzon, Philippines (1968)	Philippines 42 PHI	######
Lithocarpus aspericupulus (Markgr.) Rehder	3281 Herbarium	Vink 15331 (L), Vogelkop Peninsula, Papua New Guinea (1962)	New Guinea 43 NWG	######
Lithocarpus atjehensis Hatus. Ex Soepadmo	3259 Herbarium	De Wilde & de Wilde-Duyfjes 16001 (L), Sumatra, Indonesia (1975)	N. Sumatra 42 SUM	######
Lithocarpus atjehensis Hatus. Ex Soepadmo	3260 Herbarium	De Wilde & Wilde-Duyfjes 16128 (L), Sumatra, Indonesia (1975)	N. Sumatra 42 SUM	######
Lithocarpus auriculatus (Hickel & A. Camus) A. Camus	1977 Silica	Fuse K. L2 (KYO), Laos	Indo-China 41 LAO MYA THA VIE	######
Lithocarpus bancanus (Scheff.) Rehder	793 Silica	Chamchunroon 5153 (BKF), Chiang Mai, Thailand (2011)	Thailand to W. Malesia 41 THA 42 BOR MLY SUM	######
Lithocarpus bennettii (Miq.) Rehder	980 Silica	Strijk 980 (BGT), Nee Soon, Singapore (2012)	Pen. Thailand to W. Malesia 41 THA 42 BOR MLY SUM	######
Lithocarpus cantleyanus (King ex Hook f.) Rehder	977 Silica	Strijk 977 (BGT), Singapore (2012)	Myanmar to Malaya, Borneo 41 MYA THA 42 BOR MLY	######
Lithocarpus caudatifolius (Merr.) Rehder	1367 Silica	Strijk 1367 (BGT), Sabah, Malaysia (2014)	Borneo to N. Sulawesi 42 BOR PHI SUL	######
Lithocarpus dasystachyus (Miq.) Rehder	1727 Silica	Strijk 1727 (BGT), Borneo (2014)	Borneo 42 BOR	######
Lithocarpus edulis (Makino) Nakai	3140 Herbarium	Fujii 8676 (L), Tsushima, Japan (2001)	C. & S. Japan to Nansei-shoto 38 JAP NNS	######
Lithocarpus edulis (Makino) Nakai	1943 Silica	Tagane S. 111 (KAG), Mt. Boroishi, Japan	C. & S. Japan to Nansei-shoto 38 JAP NNS	######
Lithocarpus elephantum (Hance) A. Camus	1961 Silica	Toyama et al. 654 (KYO), Central Cardamon, Cambodia	Indo-China 41 CBD THA VIE	######
Lithocarpus ewyckii (Korth.) Rehder	984 Silica	Strijk 984 (BGT), Bukit Timah, Singapore (2012)	Malaya, Sumatra, Borneo 42 BOR MLY SUM	######
Lithocarpus formosanus (Skan) Hayata	3261 Herbarium	Liao 992 (L), Mt. Hiiran, Taiwan (1967)	Taiwan 38 TAI	######
Lithocarpus formosanus (Skan) Hayata	3262 Herbarium	Bogle et al s.n. (L), Nan-rin-shan, Taiwan (1962)	Taiwan 38 TAI	######
Lithocarpus gracilis (Korth.) Soepadmo	987 Silica	Strijk 987 (BGT), Bukit Timah, Singapore (2012)	Pen. Thailand to W. Malesia 42 BOR MLY SUM	######
Lithocarpus imperialis (Seemen) Markgr.	3345 Herbarium	Frodin & Hay 6594 (L), Central Province, New Guinea (1978)	New Guinea 43 NWG	######
Lithocarpus indutus (Blume.) Rehder	2661 Herbarium	Yahara et al. G48 (KYO), Gede, Java, Indonesia	Jawa, C. Sulawesi 42 JAW SUL	######
Lithocarpus indutus (Blume.) Rehder	2666 Silica	Yahara et al. IJ963 (KYO) Gede, Java, Indonesia	Jawa, C. Sulawesi 42 JAW SUL	######

Lithocarpus kawakamii (Hayata) Hayata	3461 Silica	Strijk 3461 (BGT), Arboretum de Passadou, France (2016)	Taiwan 38 TAI	######
Lithocarpus lauterbachii (Seemen) Markgr.	3053 Herbarium	Allison s.n. (L), Morobe, Papua New Guinea (1998)	New Guinea 43 NWG	######
Lithocarpus lucidus (Roxb.) Rehder	986 Silica	Strijk 986 (BGT), Bukit Timah, Singapore (2012)	SE. Thailand to W. Malesia 41 THA 42 BOR MLY SUM	######
Lithocarpus luzoniensis (Merr.) Rehder	3346 Herbarium	Jacobs s.n. (L), Luzon, Philippines (1968)	Philippines (Luzon) 42 PHI	######
Lithocarpus megacarpus Soepadmo	3298 Herbarium	Womersley NGF19331 (L), Morobe, Papua New Guinea (1964)	New Guinea 43 NWG	######
Lithocarpus menadoensis (Koord.) Soepadmo	3355 Herbarium	Brickpracht s.n. (L), Celebes. Indonesia (1931)	Sulawesi 42 SUL	######
Lithocarpus mindanaensis (Elmer) Rehder	3300 Herbarium	Britton 423 (L), Bukidnon, Philippines (1952)	Philippines (Mindanao, Leyte) 42 PHI	######
Lithocarpus orbicarpus Strijk	1234 Silica	Chamchunroon 5823 (BKF), Phang Nga, Thailand (2013)	Thailand 41 THA	######
Lithocarpus philippinensis (A.DC.) Rehder	3062 Herbarium	Soejarto et al 8072 (L), Leyte, Philippines (1993)	Philippines 42 PHI	######
Lithocarpus philippinensis (A.DC.) Rehder	3155 Herbarium	Co 3789 (L), Luzon, Philippines (1993)	Philippines 42 PHI	######
Lithocarpus pierrei (Hickel & A. Camus) A. Camus	1958 Silica	Tagane et al. 5514 (KYO), Mt. Bokor, Cambodia	Indo-China 41 CBD THA VIE	######
Lithocarpus pseudomoluccus (Blume) Rehder	2663 Silica	Yahara et al. IJ881 (KYO), Gede, Java, Indonesia	Sumatra to Jawa 42 JAW SUM	######
Lithocarpus pseudomoluccus (Blume) Rehder	2664 Silica	Yahara et al. IJ446 (KYO), Gede, Java, Indonesia	Sumatra to Jawa 42 JAW SUM	######
Lithocarpus rassa (Miq.) Rehder	993 Silica	Sirimongkol 370 (BKF), Phethchabun, Thailand (2012)	Pen. Thailand to W. Malesia 41 THA 42 BOR MLY SUM	######
Lithocarpus rassa (Miq.) Rehder	1133 Silica	Strijk 1133 (BKF), Chon Buri, Thailand (2013)	Pen. Thailand to W. Malesia 41 THA 42 BOR MLY SUM	######
Lithocarpus recurvatus Barnett	939 Silica	Sirimongkol 430 (BKF), Khamphaengphet, Thailand (2012)	Indo-China 41 LAO THA VIE	######
Lithocarpus recurvatus Barnett	971 Silica	Sirimongkol 462 (BKF), Nakhon Nayok, Thailand (2012)	Indo-China 41 LAO THA VIE	######
Lithocarpus ruminatus Sopeadmo	1732 Silica	Strijk 1732 (BGT), Sabah, Borneo (2014)	Borneo (Sabah) 42 BOR	######
Lithocarpus sogerensis (S. Moore) Markgr. Ex A. Camus	3366 Herbarium	Sayers NGF21578 (L), Morobe, Papua New Guinea (1965)	NE. New Guinea 43 NWG	######
Lithocarpus solerianus (Vidal) Rehder	3350 Herbarium	Lagrimas & Mabean 40617 (L), Philippines (1960)	Philippines 42 PHI	######
Lithocarpus sootepensis (Craib) A. Camus	1041 Silica	Chamchumroon 5458 (BKF), Chiang Mai, Thailand (2012)	N. Thailand, Pen. Malaysia (Kedah) 41 THA 42 MLY	######
Lithocarpus sootepensis (Craib) A. Camus	1044 Silica	Chamchunroon 5461, (BKF) Chiang Mai, Thailand (2012)	N. Thailand, Pen. Malaysia (Kedah) 41 THA 42 MLY	######
Lithocarpus sootepensis (Craib) A. Camus	1034 Silica	Chamchunroon 5451, (BKF) Chiang Mai, Thailand (2012)	N. Thailand, Pen. Malaysia (Kedah) 41 THA 42 MLY	######
Lithocarpus sulitii Soepadmo	3333 Herbarium	Pancho 2184 (L), Laguna, Philippines (1962)	Philippines (Luzon, Mindanao) 42 PHI	######
Lithocarpus thomsonii (Miq.) Rehder	760 Silica	Strijk 760 (BGT), Queen Sirikit Botanical Garden, Thailand (2011)	Assam to SC. China and Indo-China 36 CHC CHT 40 ASS BAN 41 LAO MYA THA VIE	######
Lithocarpus thomsonii (Miq.) Rehder	763 Silica	Strijk 763 (BGT), Queen Sirikit Botanical Garden, Thailand (2011)	Assam to SC. China and Indo-China 36 CHC CHT 40 ASS BAN 41 LAO MYA THA VIE	######

Lithocarpus truncatus (King ex Hook.f.) Rehder	904 Silica	Sirimongkol 395 (BKF), Phetchabun, Thailand (2012)	Assam to SC. China and Indo-China 36 CHC 40 ASS BAN 41 LAO MYA THA VIE	######
Lithocarpus truncatus (King ex Hook.f.) Rehder	924 Silica	Sirimongkol 415 (BKF), Chaiyaphum, Thailand (2012)	Assam to SC. China and Indo-China 36 CHC 40 ASS BAN 41 LAO MYA THA VIE	######
Lithocarpus vestitus (Hickel & A. Camus) A. Camus	888 Silica	Sirimongkol 379 (BKF), Phetchabun, Thailand (2012)	China (Yunnan) to Indo-China 36 CHC 41 CBD LAO THA VIE	######
Lithocarpus vestitus (Hickel & A. Camus) A. Camus	952 Silica	Sirimongkol 443 (BKF), Khamphaengphet, Thailand (2012)	China (Yunnan) to Indo-China 36 CHC 41 CBD LAO THA VIE	######
Lithocarpus woodii (Hance) A. Camus	3364 Herbarium	Kellman ANU1732, (L), Mindanao, Philippines (1965)	Philippines 42 PHI	######
Lithocarpus woodii (Hance) A. Camus	3365 Herbarium	Elmer 10511 (L), Mindanao, Philippines (1909)	Philippines 42 PHI	######
**Outgroups**				
Quercus annulata Sm.	Silica	Tagane et al. V4730 (KYO), Vietnam	Himalaya to N. Vietnam 36 CHC CHT 40 ASS EHM NEP WHM 41 MYA VIE	LC318796 (rbcL) LC318516(matK) MF770291(ITS)
Quercus auricoma A. Camus	Silica	Ngoc et al. V3135 (KYO), Vietnam	Vietnam 41 VIE	LC318778 (rbcL) LC318498(matK) MF770277(ITS)
Quercus austrocochinchinensis Hickel & A. Camus	Silica	Ngoc et al. V3129 (KYO), Vietnam	China (Guangxi, Yunnan) to Indo-China 36 CHC CHS 41 LAO THA VIE	LC318777(rbcL) LC318497(matK) MF770276(ITS)
Q. bambusifolia Hance	Silica	Ngoc et al. V3788 (KYO), Vietnam	China (Guangdong, Guangxi) to N. Vietnam 36 CHH CHS 41 VIE	LC318789(rbcL) LC318509(matK) MF770284(ITS)
Quercus macrocalyx Hickel & A. Camus	Silica	Tagane et al. V6457 (KYO), Vietnam	S. China to Indo-China 36 CHC CHH CHS CHT 41 LAO VIE	LC318800(rbcL) LC318520(matK) MF770294(ITS)
Quercus kerri Craib	Silica	Tagane et al. V6765 (KYO), Vietnam	SE. Bangladesh to Hainan 36 CHC CHH 40 BAN 41 LAO MYA THA VIE	LC318801(rbcL) LC318521 (matK) MF770295(ITS)
Quercus sp.	Silica	Hoang et al. V5101 (DLU, FU), Vietnam	Hainan to W. Malesia 36 CHH 40 ASS BAN 41 LAO MYA THA VIE 42 BOR JAW MLY SUM	######
Quercus poilanei Hickel & A. Camus	Silica	Yahara et al. V2986 (KYO), Vietnam	China (Guangxi) to N. Indo-China 36 CHS 41 THA VIE	LC318774(rbcL) LC318494 (matK) MF770273(ITS)
