# Supplementary material for: Museomics for reconstructing historical floristic exchanges: Divergence of stone oaks across Wallacea
Source: PLoS One. 2020 May 22;15(5):e0232936. doi: 10.1371/journal.pone.0232936 (PMC7244142; doi:10.1371/journal.pone.0232936)
Supplement: S2 Table — Results of the Tukey HSD comparisons tests for the 4 zones configuration (a) or 6 zones configuration (b); **: p<0.01 significance threshold. (PDF) [file pone.0232936.s002.pdf]

Supplementary Table S2a. Results of the Tukey HSD comparisons tests for the 4 zones scenario; **: $p<0.01$ significance threshold.

	**zone1**	**zone2**	**zone3**	**zone4**
**zone1**				
**zone2**	0.0004518 **			
**zone3**	0.0038006 **	0.9455306		
**zone4**	0.7420784	0.0000041 **	0.0000611 **	

Supplementary Table S2b. Results of the Tukey HSD comparisons tests for the 6 zones scenario; **: $p<0.01$ significance threshold.

	**zone1**	**zone2**	**zone3**	**zone4**	**zone5**	**zone6**
**zone1**						
**zone2**	0.0000001 **					
**zone3**	$<10^{16}$ **	0.9999991				
**zone4**	0.9526068	$<10^{16}$ **	$<10^{16}$ **			
**zone5**	0.8288091	$<10^{16}$ **	$<10^{16}$ **	0.999384		
**zone6**	0.6591158	$<10^{16}$ **	$<10^{16}$ **	0.9885456	0.9997206	
