# Supplementary figures and images for: Museomics for reconstructing historical floristic exchanges: Divergence of stone oaks across Wallacea
Source: PLoS One. 2020 May 22;15(5):e0232936. doi: 10.1371/journal.pone.0232936 (PMC7244142; doi:10.1371/journal.pone.0232936)

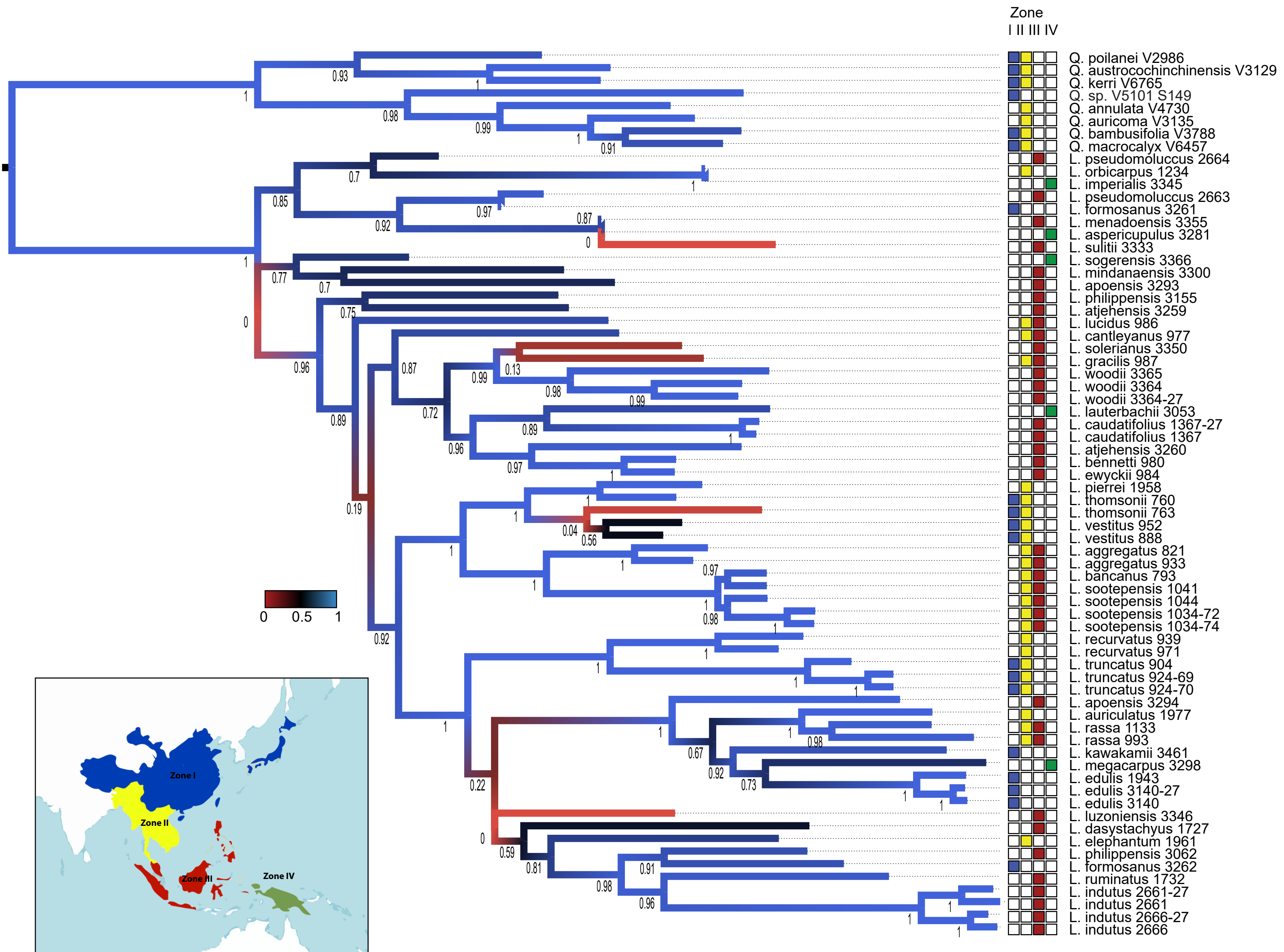

Supplement: S1 Fig — SH-like nodes support values indicated on nodes. Color of the branches indicate support values. Geographic range indicated at the tips corresponding to the inset map (see text for details). (PDF) [file pone.0232936.s003.pdf]

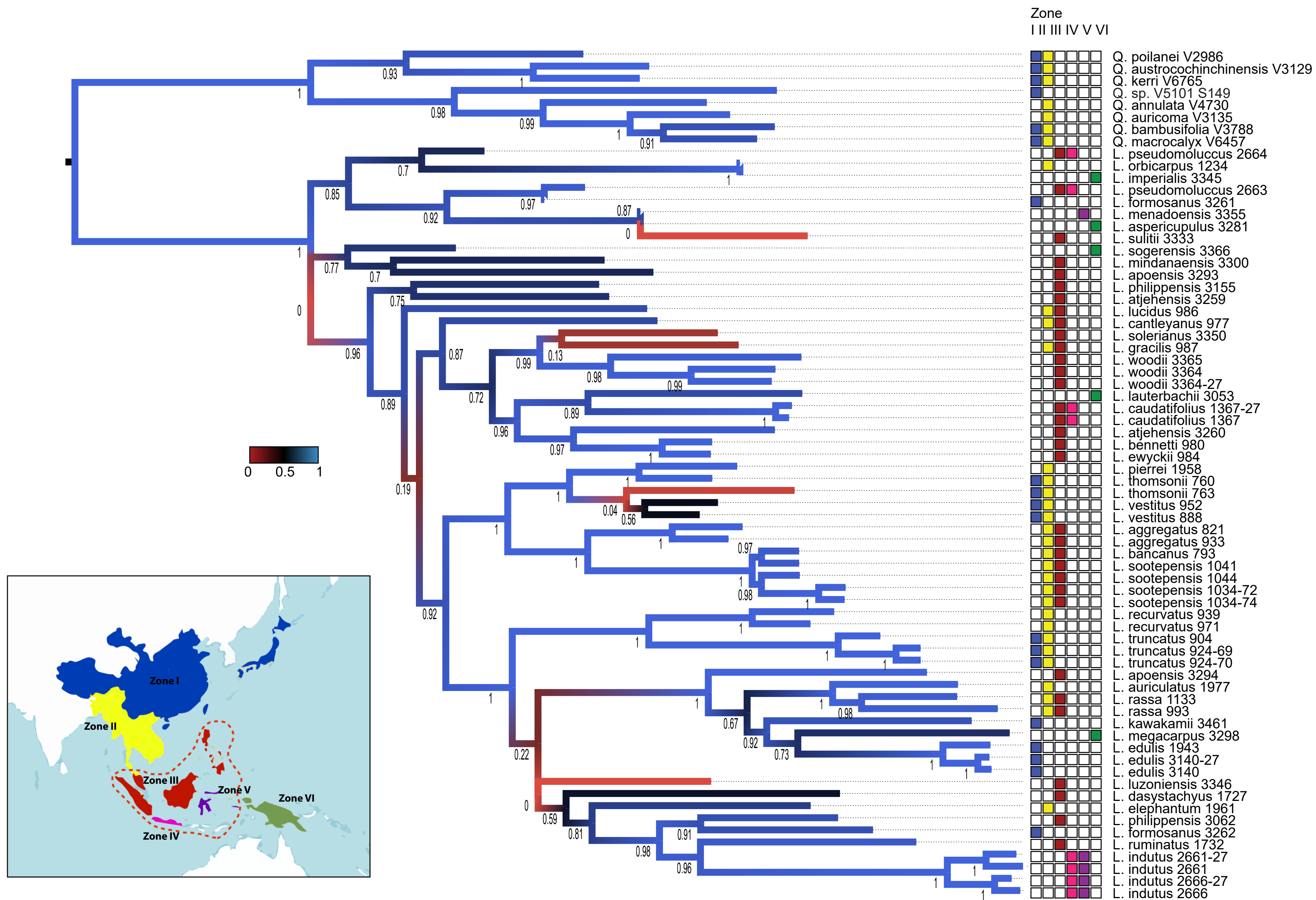

Supplement: S2 Fig — SH-like nodes support values indicated on nodes. Color of the branches indicate support values. Geographic range indicated at the tips corresponding to the inset map (see text for details). (PDF) [file pone.0232936.s004.pdf]

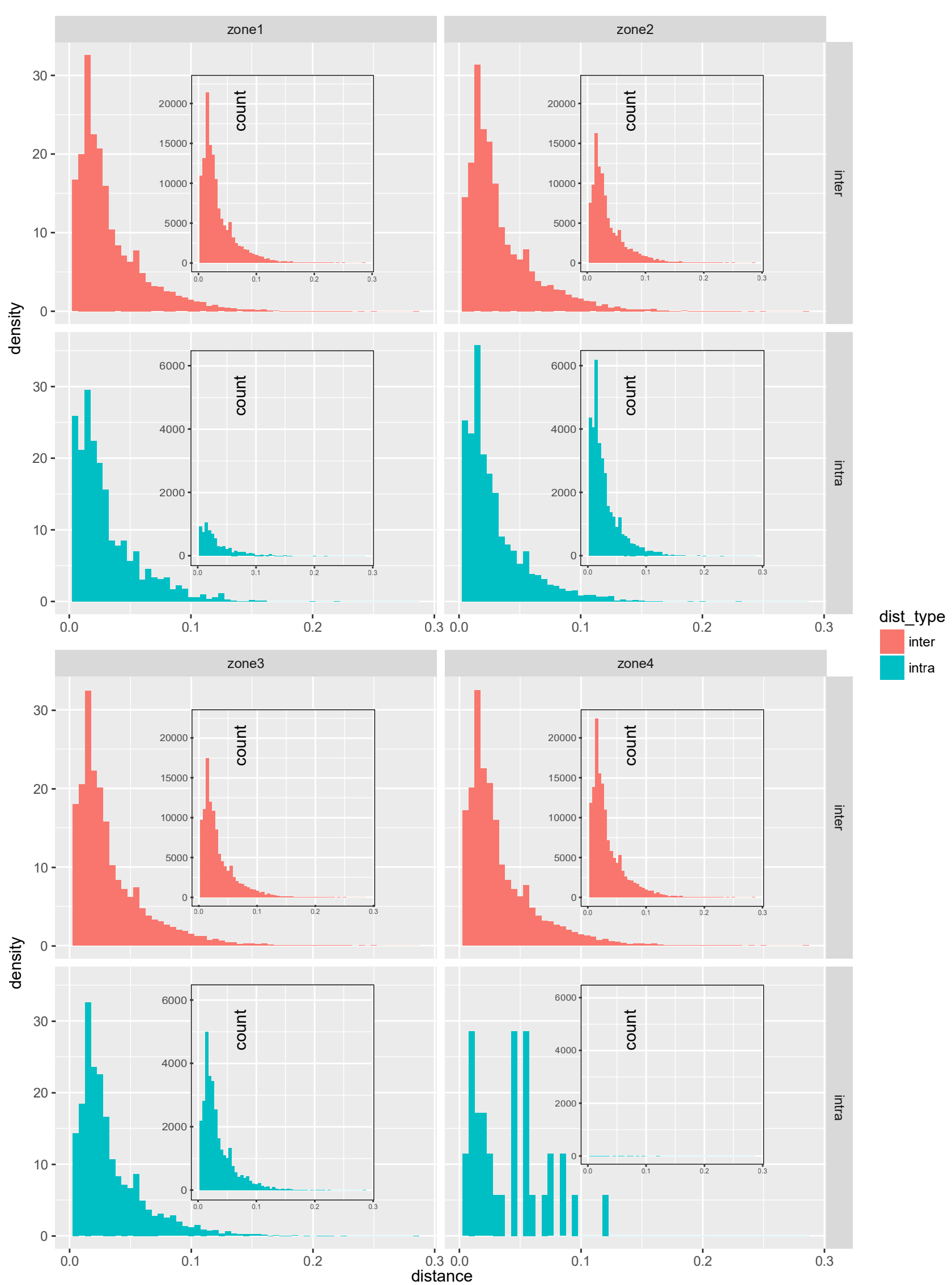

Supplement: S3 Fig — Density histogram of inter- (red) and intra- (blue) zone distances according to the 4 zones configuration. For each plot the corresponding count histogram is plotted as inset. (PDF) [file pone.0232936.s005.pdf]

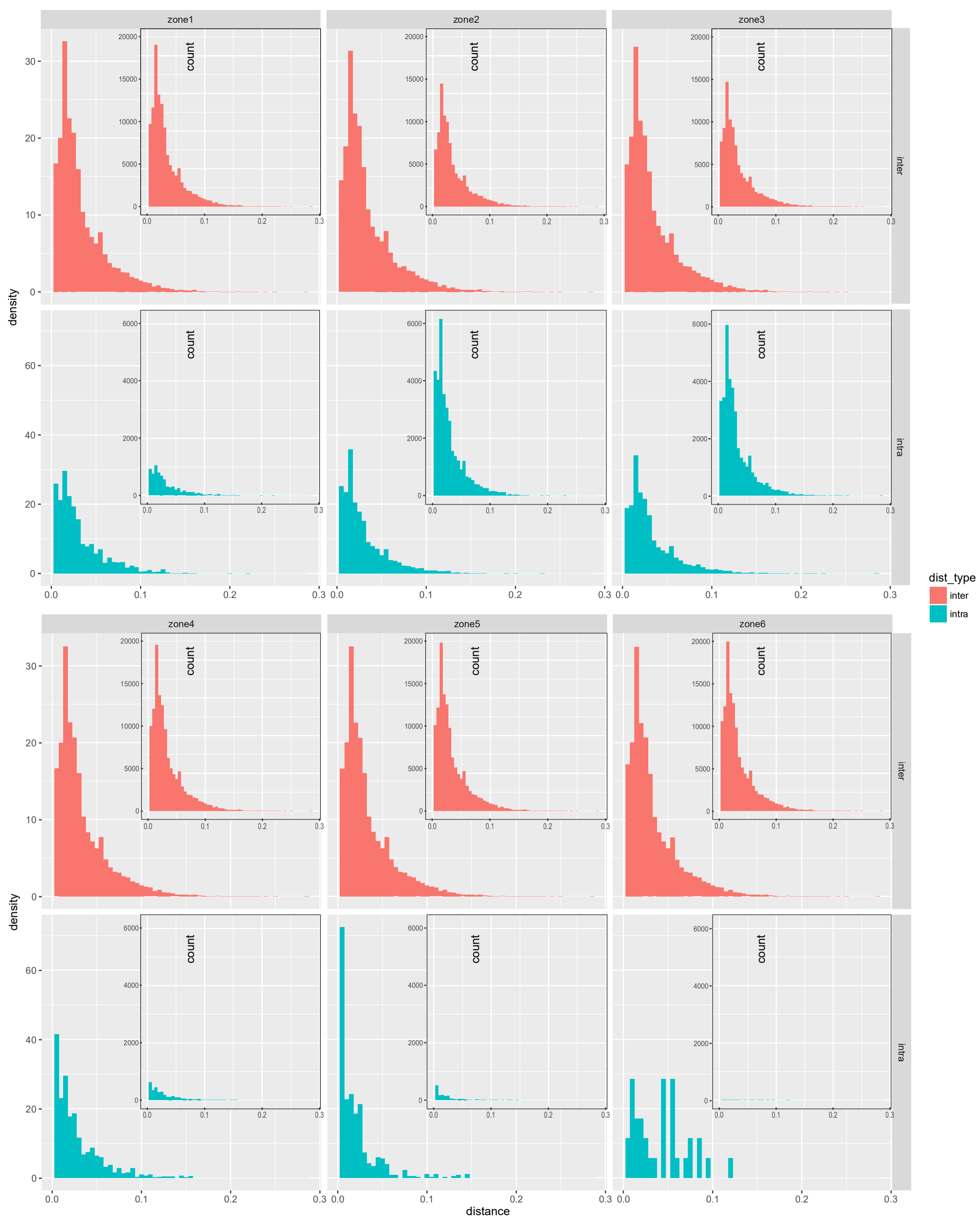

Supplement: S4 Fig — Density histogram of inter- (red) and intra- (blue) zone distances according to the 6 zones configuration. For each plot the corresponding count histogram is plotted as inset. (PDF) [file pone.0232936.s006.pdf]

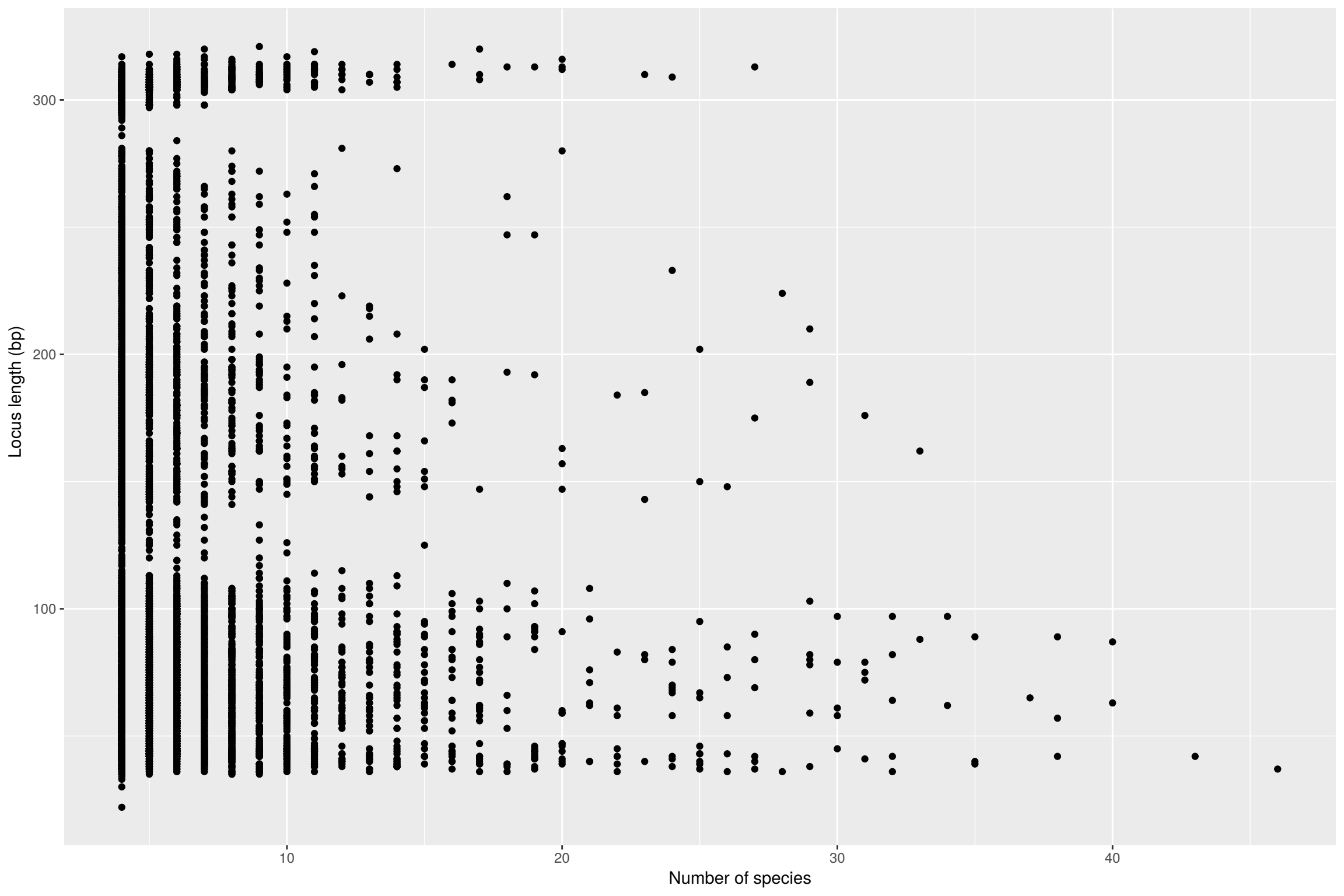

Supplement: S5 Fig — Each point represents one reconstructed locus. Horizontal axis: number of species assembled for a given locus; vertical axis: locus lengths. (PDF) [file pone.0232936.s007.pdf]

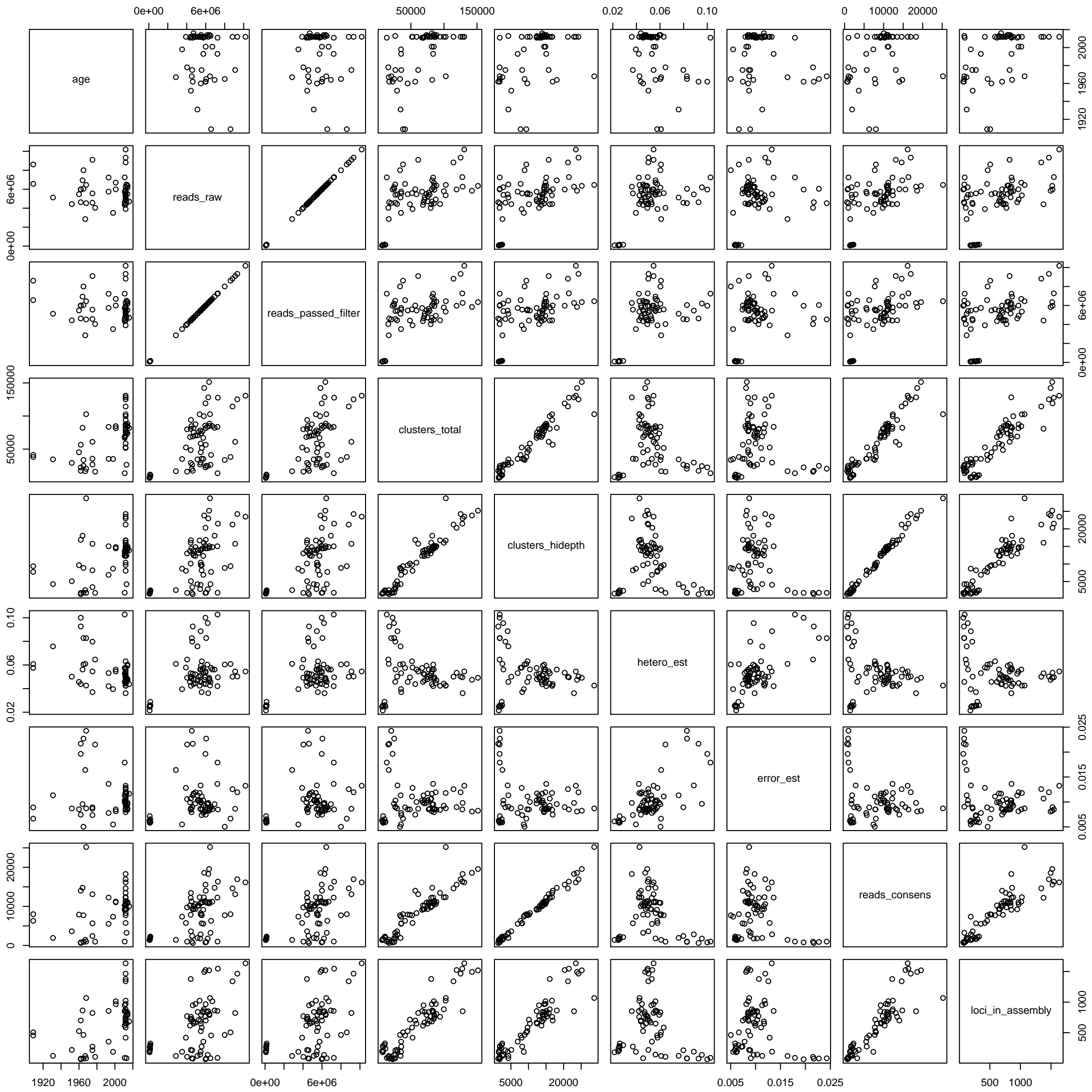

Supplement: S6 Fig — Statistics are derived from ipyrad outputs. Age: calendar year of collection of the samples; reads raw: number of generated Illumina reads used as input for the loci reconstruction for a given species; reads passed filter: number of reads after ipyrad filtering steps; clusters total: number of clusters assembled for a given species; clusters hidepth: number of clusters with a assembly depth > 5; hetero est: heterozygosity estimate for each sample; error est: error rate estimate for each sample; reads consens: number of reads from a given species used to generate the consensus sequence of a loci; loci in assembly: final number of loci reconstructed for a given species. (PDF) [file pone.0232936.s008.pdf]

A

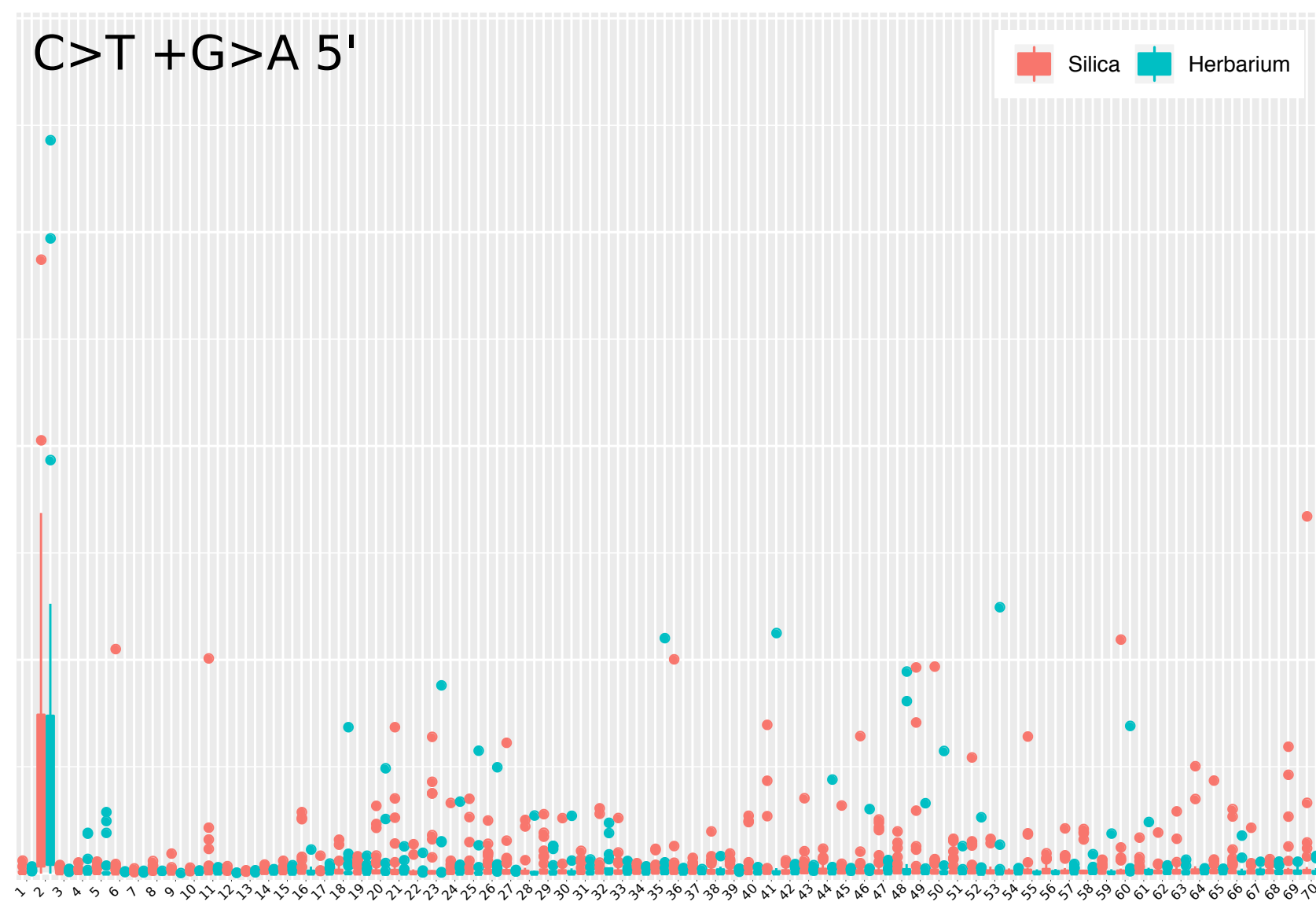

B

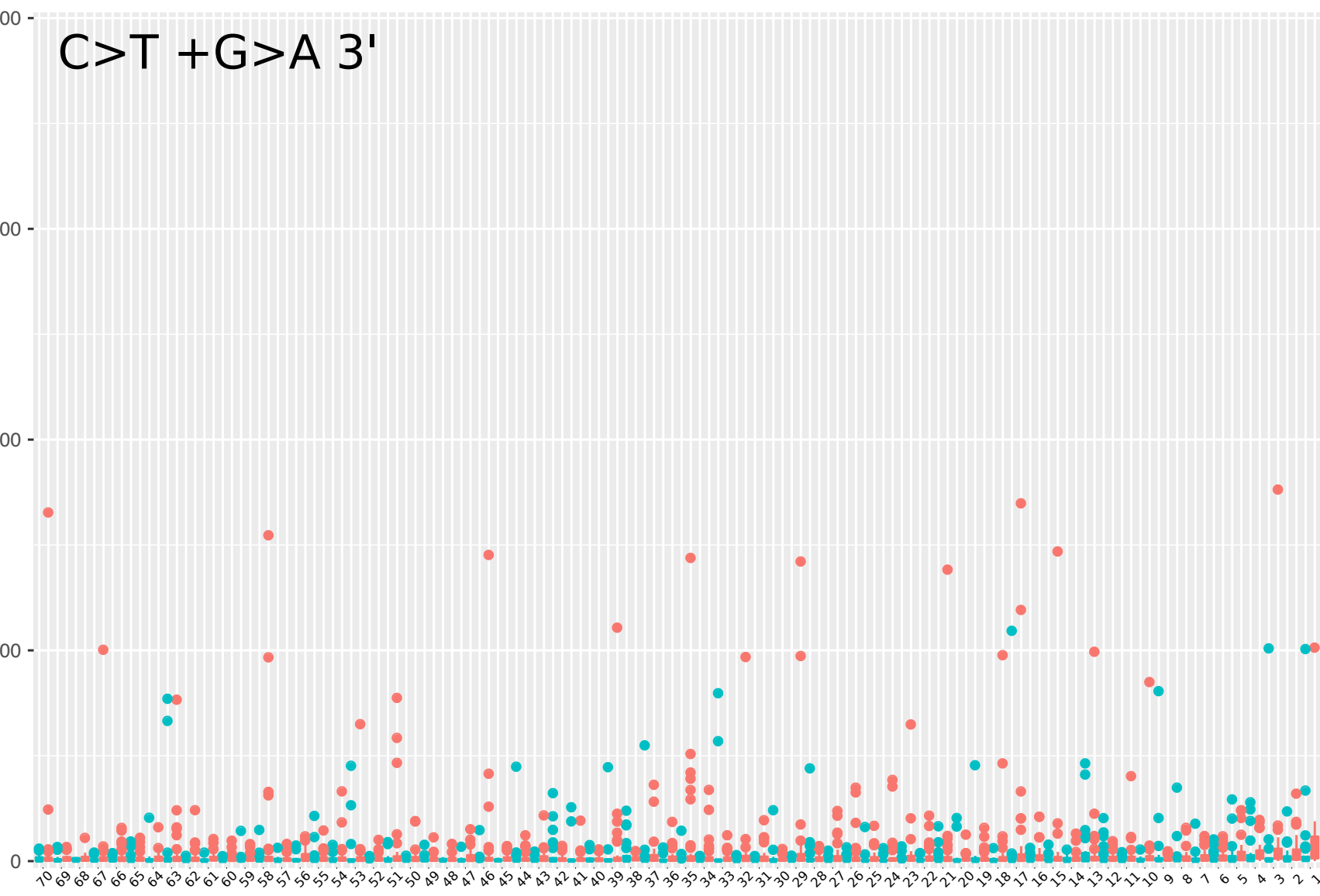

Supplement: S8 Fig — Patterns were obtained by using MapDamage v. 2.0.6. Y-axis denotes the number of reads containing a nucleotide change from the reference sequence, and x -axis denotes position along the DNA fragment. A) misincorporation patterns at 5’ ends for each sample; B) misincorporation patterns at 3’ ends for each sample. (PDF) [file pone.0232936.s010.pdf]

A

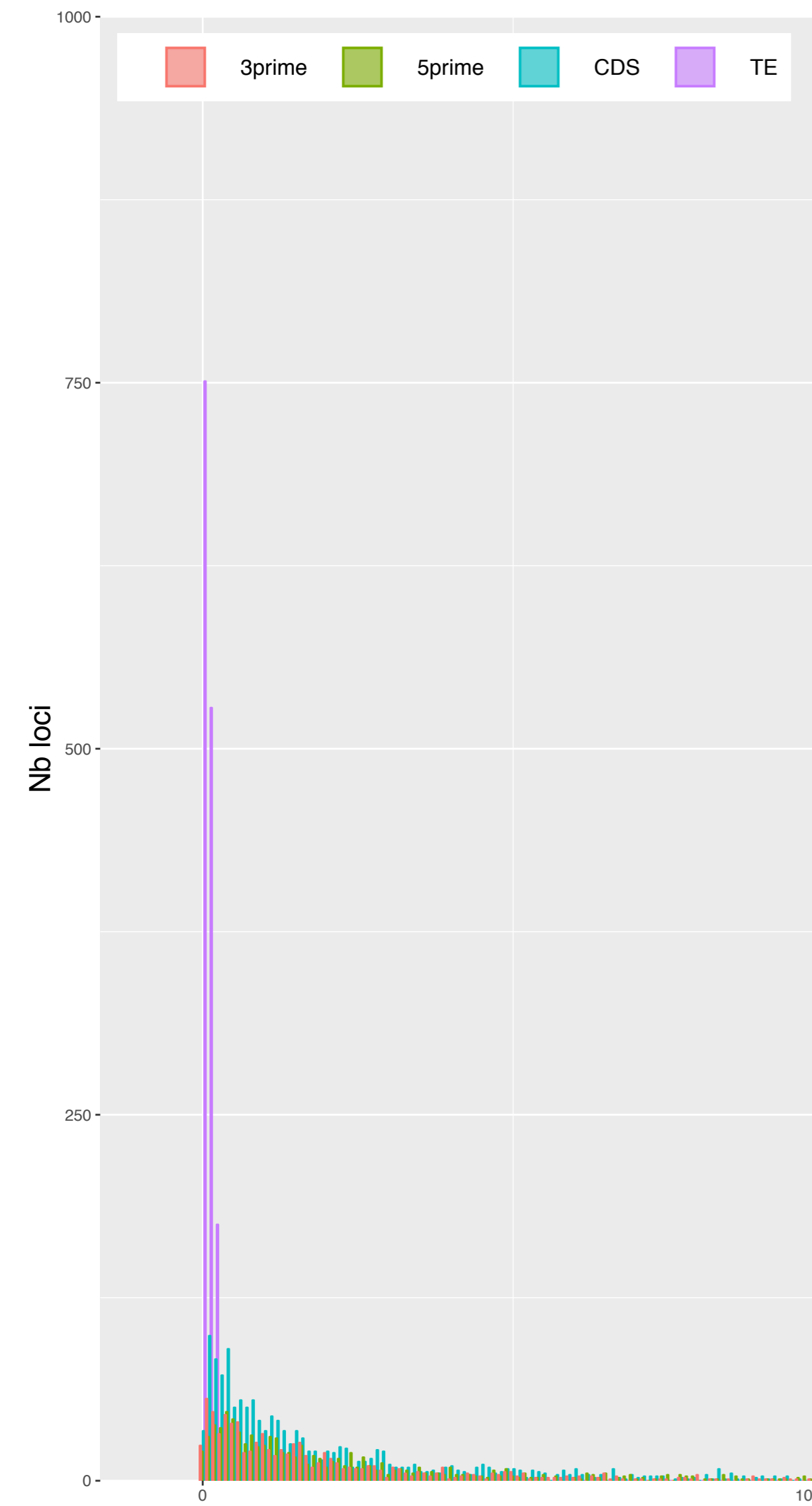

B

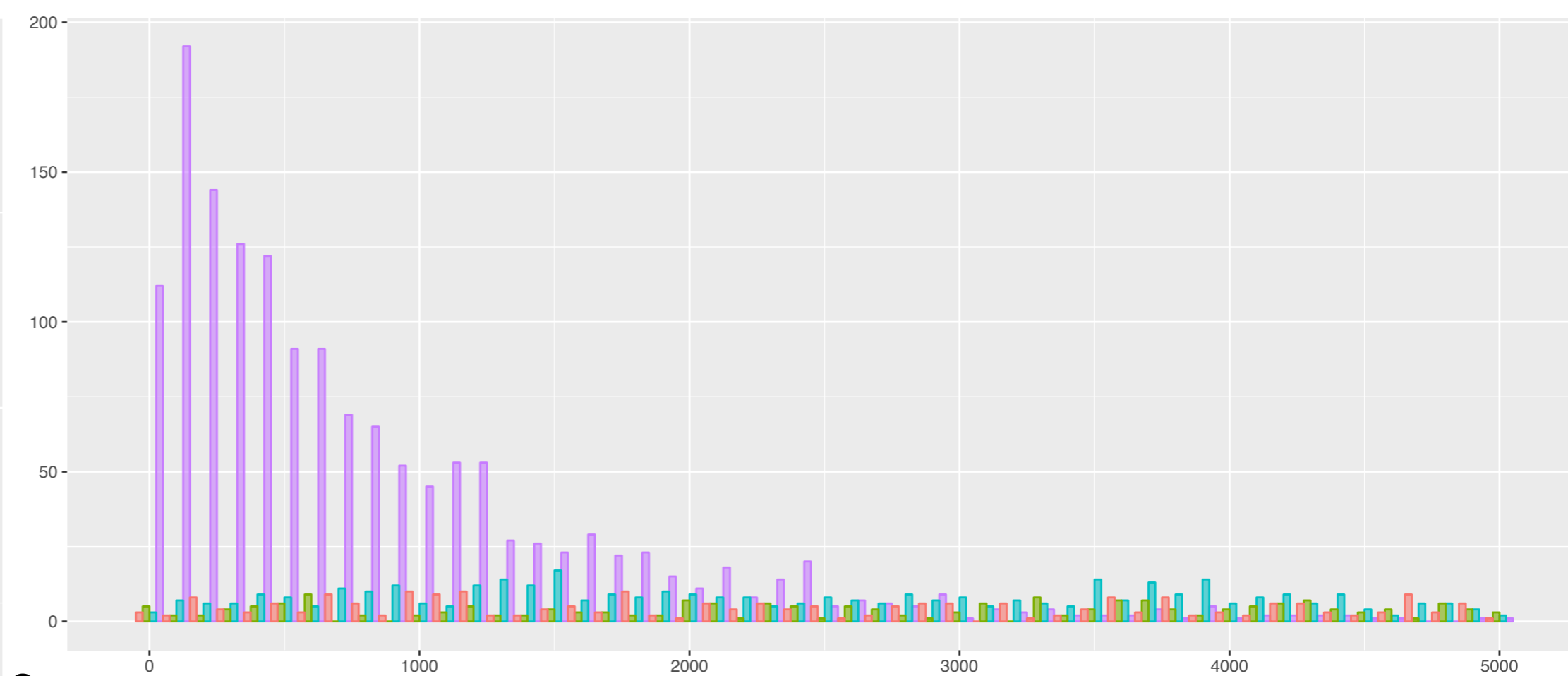

C

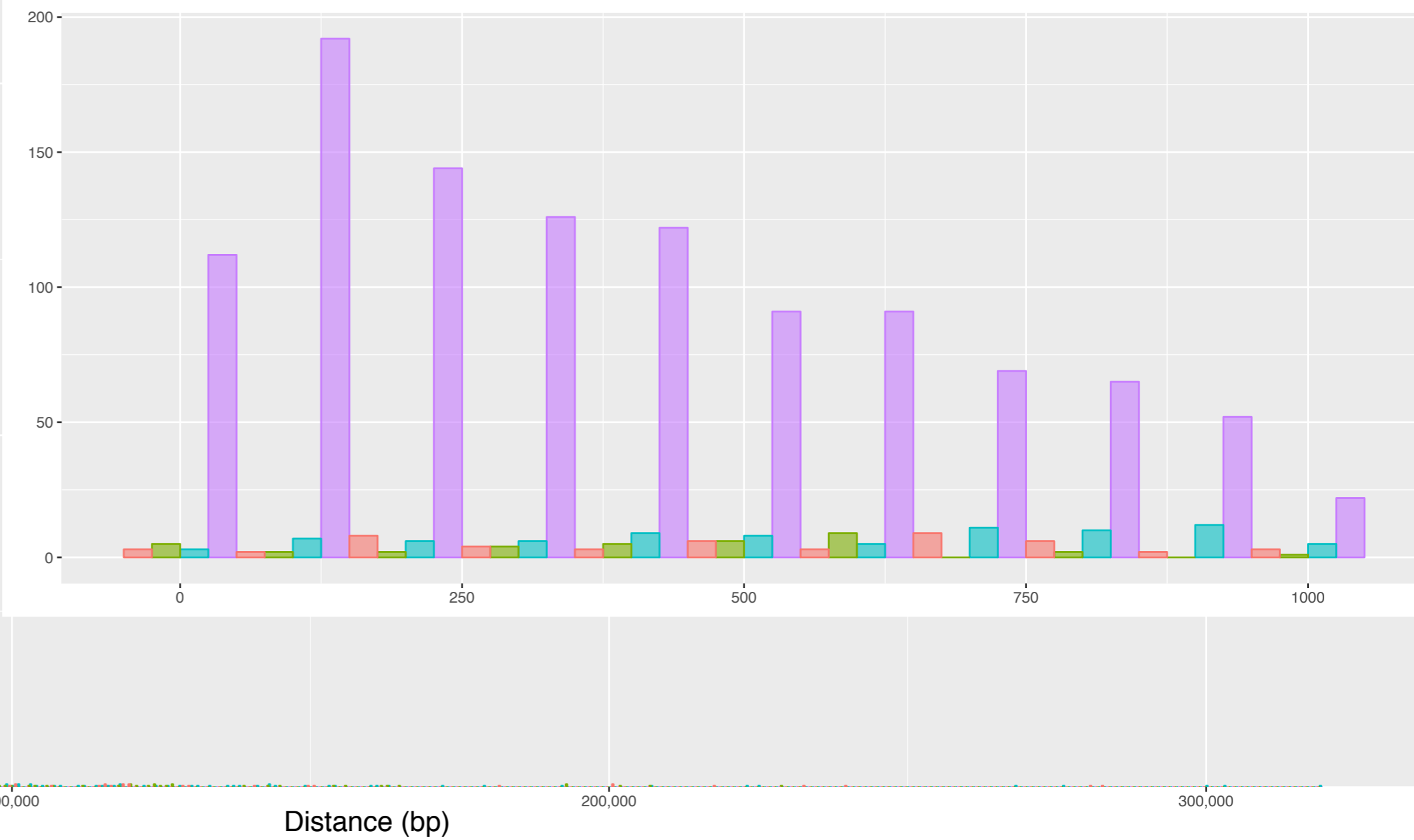

Supplement: S9 Fig — TE: Transposable Elements; 5prime: five prime Untranslated Transcribed Region; 3prime: three prime Untranslated Transcribed Region; CDS: Coding Regions. A) Distribution for all MIGseq loci; B) Distribution for MIGseq loci located closer than 5kb from a genomic feature; C) Distribution for MIGseq loci located closer than 1,000bp from a genomic feature. (PDF) [file pone.0232936.s011.pdf]

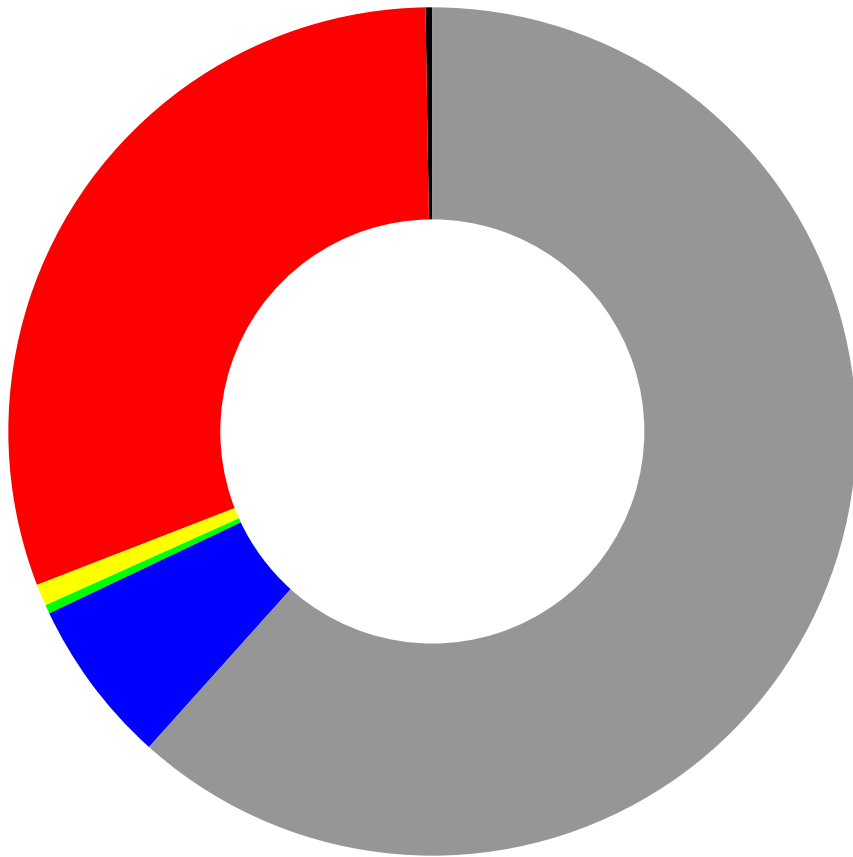

- not mapped
- in TE
- in 5' UTR
- in 3'UTR
- in CDS
- others

Supplement: S10 Fig — TE: Transposable Elements; 5’ UTR: five prime Untranslated Transcribed Region; 3’ UTR: three prime Untranslated Transcribed Region; CDS: Coding Regions; Others: not found in annotations from the PM1N oak genome. (PDF) [file pone.0232936.s012.pdf]
