# Supplementary material for: Museomics for reconstructing historical floristic exchanges: Divergence of stone oaks across Wallacea
Source: PLoS One. 2020 May 22;15(5):e0232936. doi: 10.1371/journal.pone.0232936 (PMC7244142; doi:10.1371/journal.pone.0232936)
Supplement: S7 Fig — Patterns were obtained by using MapDamage v. 2.0.6. Y-axis denotes the number of reads containing a nucleotide change from the reference sequence, and x -axis denotes position along the DNA fragment. A) misincorporation patterns at 5’ ends for each sample; B) misincorporation patterns at 3’ ends for each sample. (PDF) [file pone.0232936.s009.pdf]

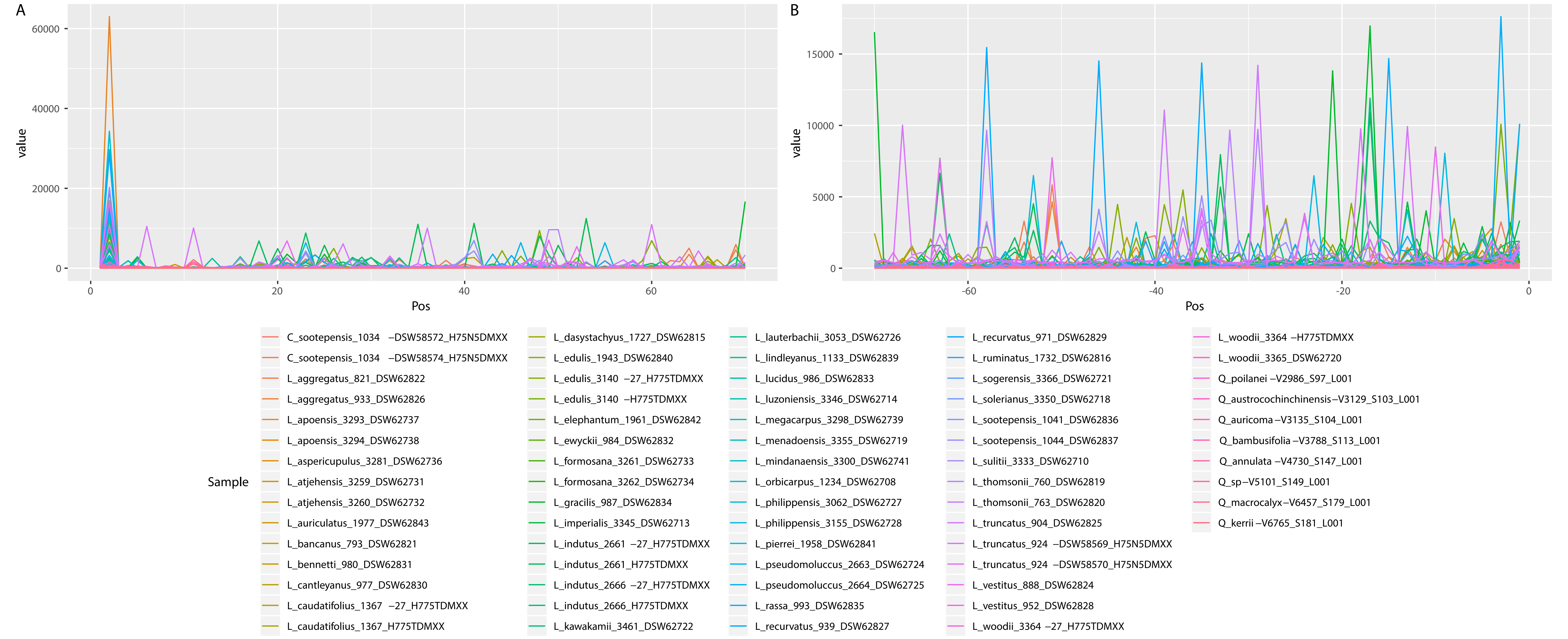
A
B
value
Pos
60000
40000
20000
0
20
40
60
15000
10000
5000
-60
-40
-20
Sample
C_sootepensis_1034 −DSW58572_H75N5DMXX
C_sootepensis_1034 −DSW58574_H75N5DMXX
L_aggregatus_821_DSW62822
L_aggregatus_933_DSW62826
L_apoensis_3293_DSW62737
L_apoensis_3294_DSW62738
L_aspericupulus_3281_DSW62736
L_atjehensis_3259_DSW62731
L_atjehensis_3260_DSW62732
L_auriculatus_1977_DSW62843
L_bancanus_793_DSW62821
L_bennetti_980_DSW62831
L_cantleyanus_977_DSW62830
L_caudatifolius_1367 −27_H775TDMXX
L_caudatifolius_1367_H775TDMXX
L_dasystachyus_1727_DSW62815
L_edulis_1943_DSW62840
L_edulis_3140 −27_H775TDMXX
L_edulis_3140 −H775TDMXX
L_elephantum_1961_DSW62842
L_ewyckii_984_DSW62832
L_formosana_3261_DSW62733
L_formosana_3262_DSW62734
L_gracilis_987_DSW62834
L_imperialis_3345_DSW62713
L_indutus_2661 −27_H775TDMXX
L_indutus_2661_H775TDMXX
L_indutus_2666 −27_H775TDMXX
L_indutus_2666_H775TDMXX
L_kawakamii_3461_DSW62722
L_lauterbachii_3053_DSW62726
L_lindleyanus_1133_DSW62839
L_lucidus_986_DSW62833
L_luzoniensis_3346_DSW62714
L_megacarpus_3298_DSW62739
L_menadoensis_3355_DSW62719
L_mindanaensis_3300_DSW62741
L_orbicarpus_1234_DSW62708
L_philippensis_3062_DSW62727
L_philippensis_3155_DSW62728
L_pierrei_1958_DSW62841
L_pseudomoluccus_2663_DSW62724
L_pseudomoluccus_2664_DSW62725
L_rassa_993_DSW62835
L_recurvatus_939_DSW62827
L_recurvatus_971_DSW62829
L_ruminatus_1732_DSW62816
L_sogerensis_3366_DSW62721
L_solerianus_3350_DSW62718
L_sootepensis_1041_DSW62836
L_sootepensis_1044_DSW62837
L_sulitii_3333_DSW62710
L_thomsonii_760_DSW62819
L_thomsonii_763_DSW62820
L_truncatus_904_DSW62825
L_truncatus_924 −DSW58569_H75N5DMXX
L_truncatus_924 −DSW58570_H75N5DMXX
L_vestitus_888_DSW62824
L_vestitus_952_DSW62828
L_woodii_3364 −27_H775TDMXX
L_woodii_3364 −H775TDMXX
L_woodii_3365_DSW62720
Q_poilanei −V2986_S97_L001
Q_austrocochinchinensis−V3129_S103_L001
Q_auricoma −V3135_S104_L001
Q_bambusifolia −V3788_S113_L001
Q_annulata −V4730_S147_L001
Q_sp−V5101_S149_L001
Q_macrocalyx−V6457_S179_L001
Q_kerrii −V6765_S181_L001
